# CasDinG is a 5′-3′ dsDNA and RNA/DNA helicase with three accessory domains essential for type IV CRISPR immunity

**DOI:** 10.1093/nar/gkad546

**Published:** 2023-07-03

**Authors:** Hannah Domgaard, Christian Cahoon, Matthew J Armbrust, Olivine Redman, Alivia Jolley, Aaron Thomas, Ryan N Jackson

**Affiliations:** Department of Chemistry and Biochemistry, Utah State University, Logan, UT, USA; Department of Chemistry and Biochemistry, Utah State University, Logan, UT, USA; Department of Chemistry and Biochemistry, Utah State University, Logan, UT, USA; Department of Chemistry and Biochemistry, Utah State University, Logan, UT, USA; Department of Chemistry and Biochemistry, Utah State University, Logan, UT, USA; Center for Integrated Biosystems, Utah State University, Logan, UT, USA; Department of Chemistry and Biochemistry, Utah State University, Logan, UT, USA

## Abstract

CRISPR-associated DinG protein (CasDinG) is essential to type IV-A CRISPR function. Here, we demonstrate that CasDinG from *Pseudomonas aeruginosa strain 83* is an ATP-dependent 5′-3′ DNA translocase that unwinds double-stranded (ds)DNA and RNA/DNA hybrids. The crystal structure of CasDinG reveals a superfamily 2 helicase core of two RecA-like domains with three accessory domains (N-terminal, arch, and vestigial FeS). To examine the *in vivo* function of these domains, we identified the preferred PAM sequence for the type IV-A system (5′-GNAWN-3′ on the 5′-side of the target) with a plasmid library and performed plasmid clearance assays with domain deletion mutants. Plasmid clearance assays demonstrated that all three domains are essential for type IV-A immunity. Protein expression and biochemical assays suggested the vFeS domain is needed for protein stability and the arch for helicase activity. However, deletion of the N-terminal domain did not impair ATPase, ssDNA binding, or helicase activities, indicating a role distinct from canonical helicase activities that structure prediction tools suggest involves interaction with dsDNA. This work demonstrates CasDinG helicase activity is essential for type IV-A CRISPR immunity as well as the yet undetermined activity of the CasDinG N-terminal domain.

## Graphical Abstract

**Graphical Abstract:**
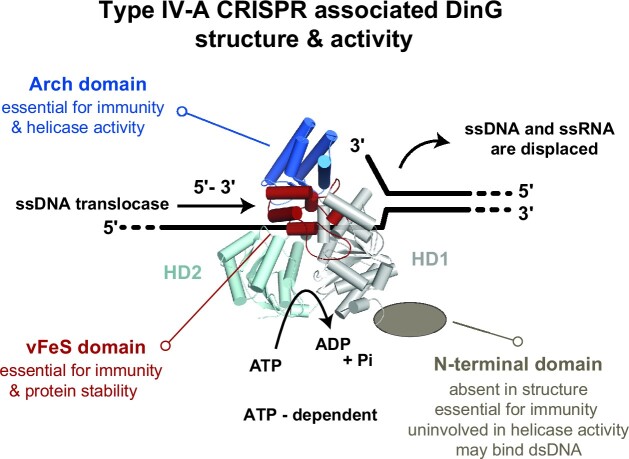

## INTRODUCTION

**Figure 1.**
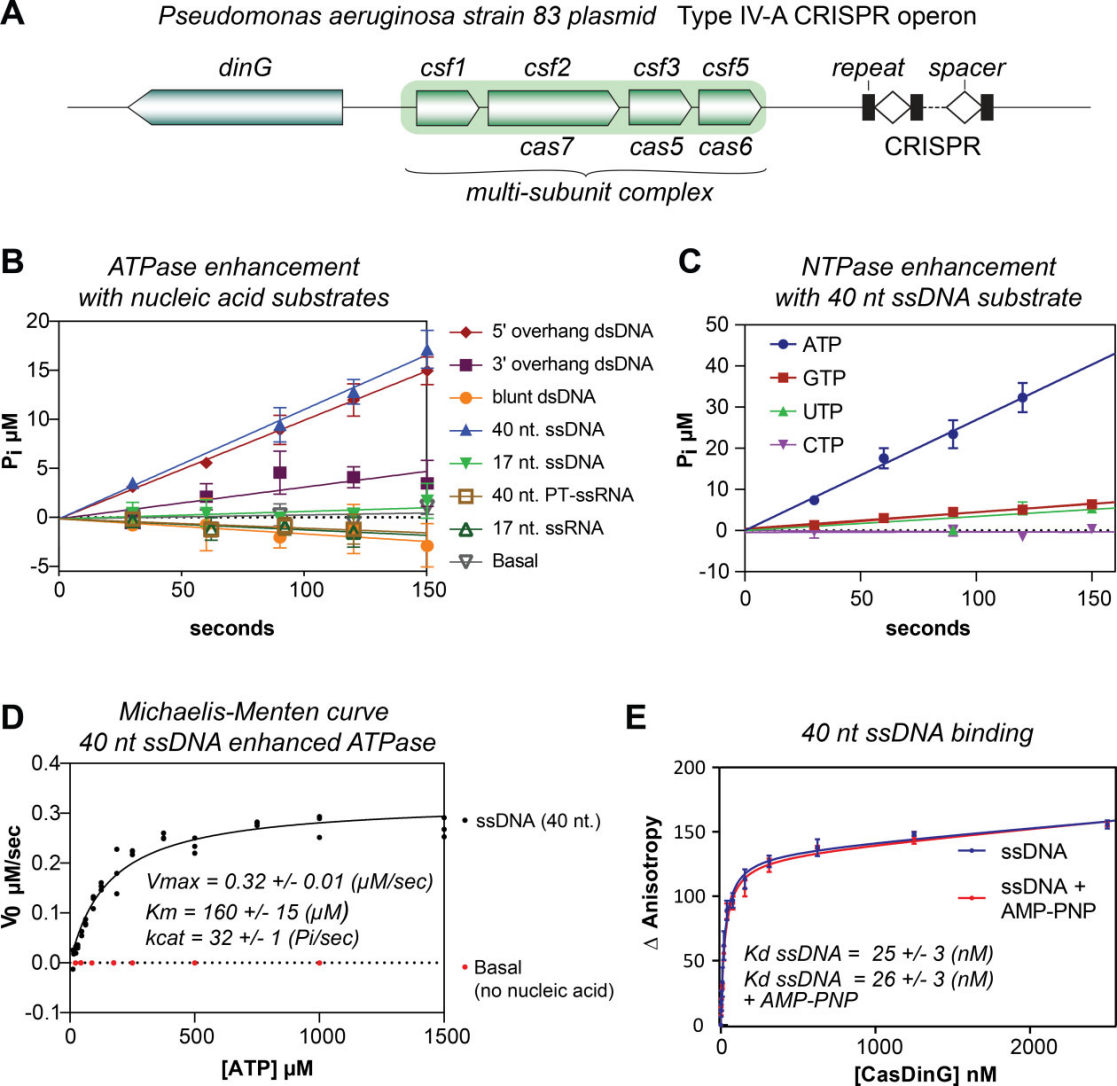
CasDinG is a nucleic acid-dependent ATPase. (**A**) Type IV-A CRISPR operon from *P. aeruginosa strain 83 plasmid*. (**B**) V_0_ plots depicting inorganic phosphate (P_i_) production via ATP hydrolysis with 10 nM CasDinG in the presence of 100 nM of different nucleic acid substrates and 600 μM ATP. (**C**) V_0_ plot depicting P_i_ phosphate production via NTP hydrolysis in the presence of 10 nM CasDinG and 600 μM NTP. (**D**) Michaelis-Menten curve depicting rates of P_i_ phosphate production at various ATP concentrations in the presence of 100 nM 40 nt ssDNA substrate with 10 nM CasDinG or without any ssDNA (basal). (**E**) Fluorescence anisotropy curve of CasDinG binding to a 40 nt. 3′- FAM labeled ssDNA with and without AMP-PNP.

CRISPR-Cas systems are prokaryotic immune systems that protect against mobile genetic elements, such as viruses and plasmids (1,2). Of the six known CRISPR system types, type IV systems are the least understood and display a large diversity of protein composition (3–5). Type IV-A systems rely on a multi-subunit complex consisting of a CRISPR-derived RNA (crRNA) bound by several Type IV-specific CRISPR-associated proteins (Csf1, Csf2, Csf3, Csf5/Cas6), and the CRISPR-associated DinG (CasDinG) protein to clear invasive plasmids (6–8) (Figure 1A). Mutating either the CasDinG Walker A or Walker B motifs, predicted to bind and hydrolyze ATP, impaired type IV immunity (7,8), suggesting CasDinG-mediated ATP binding and hydrolysis are essential for type IV-A function.

DinG (*damage-inducible gene G*) proteins are superfamily 2 (SF2) helicases related to eukaryotic XPD helicases involved in nucleotide excision repair (9–11). Deletion of the *dinG* gene in *E. coli* results in ultraviolet radiation sensitivity and mutations in the human XPD protein cause heritable ultrasensitivity to ultraviolet light and premature cancers (12,13). Notably, in bacteria DinG orthologs display variability in domain organization and the functional role of ATP binding and hydrolysis. For example, *E. coli* DinG is a 5′- 3′ DNA helicase that relies on ATP hydrolysis and an accessory domain containing an iron-sulfur (FeS) cluster to unwind substrates (13–15), while *S. aureus* DinG lacks an FeS cluster domain and does not unwind DNA (16). Instead, *S. aureus* DinG contains an N-terminal accessory domain with 3′-5′ exonuclease activity that is regulated by ATP binding.

CasDinG proteins are distinct from non-Cas DinG sequences (17), including the *E. coli* and *S. aureus* DinG proteins described above (Supplementary Figure S1). For example, CasDinG contains an accessory domain in the same location as the *E. coli* FeS cluster domain, but the amino acid sequence of the CasDinG domain lacks cysteines for coordinating an FeS cluster (17). Additionally, CasDinG has an N-terminal accessory domain, but it is smaller than the *S. aureus* N-terminal exonuclease domain and bioinformatic studies do not predict the domain to harbor an exonuclease activity (Supplementary Figure S1) (4,5). Thus, sequence comparisons to previously investigated DinG proteins could not fully reveal the biochemical function of CasDinG.

To better understand the function of CasDinG in type IV-A CRISPR immunity, we performed ATPase, nucleic acid binding, and helicase assays with recombinant CasDinG protein encoded on a *P. aeruginosa strain 83* extrachromosomal plasmid (NCBI ref: NZ_CP017294.1), referred to throughout the paper as CasDinG (Figure 1A). We also solved the x-ray crystal structure of CasDinG, defined the type IV-A consensus protospacer adjacent motif (PAM) with a plasmid curing library, and performed cell-based assays with single point mutations and domain deletion mutants. Our results indicate CasDinG is an ATP-dependent 5′-3′ DNA translocase capable of unwinding DNA and RNA/DNA duplexes comprised of an SF2 helicase core and three accessory domains. Plasmid curing assays indicate each of these domains (N-terminal, vFeS, and arch) is essential for type IV-A immunity. Notably, the removal of the N-terminal domain inhibited type IV-A immunity, but did not disrupt ATPase or helicase activities, indicating the N-terminal domain serves an essential function in type IV-A immunity beyond substrate unwinding. Predicted structures of the N-terminal domain suggest it adopts a fold observed in dsDNA binding proteins, indicating the N-terminal domain may interact directly with dsDNA. This work provides a structural and biochemical foundation for understanding the role of CasDinG in type IV-A CRISPR immunity and reveals that the N-terminal domain of CasDinG plays an essential but unclear role that may hold the key to understanding the elusive type IV-A immune systems.

## MATERIALS AND METHODS

### Construct generation, expression & purification of full length and domain deletion proteins

The *dinG* gene from a *P. aeruginosa strain 83* extrachromosomal plasmid (NCBI ref: WP_088922490.1 (protein sequence), NZ_CP017294.1 (plasmid sequence)) was synthesized by TWIST bioscience and cloned into a pET StrepII TEV ligation independent cloning (LIC) vector (2R-T) (7). The annotated gene begins with a non-canonical (TTG) start codon, but the open reading frame continues several amino acids upstream of the gene annotation. Thus, to ensure we were expressing the entire biologically relevant sequence we included the in-frame sequence upstream of the annotation starting with another non-canonical (TTG) start codon before reaching a stop codon. This approach added 48 DNA bases to the annotated gene and 16 additional amino acids (MKLAQGAFVDVIRIGA) to the N-terminus of the annotated amino acid sequence. Additionally, with this modification, the currently annotated TTG start codon would encode for a leucine instead of methionine.

Domain deletion constructs for recombinant expression and cell-based assays were generated using the NEBaseChanger tool to design primers and the NEB Q5 Site-directed Mutagenesis Kit (E0554). For the N-terminal domain deletion, 306 bases (encoding amino acids 2–103) were removed. For the vFeS domain deletion, bases encoding amino acids 200–263 were replaced with bases encoding three glycines. For the vFeS loop deletion, the bases encoding amino acids 227–246 were replaced with bases encoding tryptophan, aspartate, and glycine (the amino acid sequence observed in EcDinG in this region). For the arch domain deletion, bases encoding residues 351–464 were removed, directly linking amino acids 350–465.

Vectors were transformed into *E. coli* BL21 HMS174(DE3) chemically competent cells (Novagen). A colony was picked and placed into an overnight outgrowth in Luria-Bertani (LB) media at 37°C. In a 2.8 l flask, 1 l of LB medium supplemented with 1 ml of 1000× metals mix (0.1 M FeCl_3_–6H_2_O, 1 M CaCl_2_, 1 M MnCl_2_-4H_2_O, 1 M ZnSO_4_–7H_2_O, 0.2 M CoCl_2_–6H_2_O, 0.1 M CuCl_2_–2H_2_O, 0.2 M NiCl_2_–6H_2_O, 0.1 M Na_2_MoO_4_–2H_2_O, 0.1 M Na_2_SeO_3_–5H_2_O, 0.1 M H_3_BO_3_) and 1 ml of 1000× MgSO_4_ (1 M) was inoculated with 20 ml of overnight starter. Cells were grown to an optical density between 1.0 and 1.3 OD_600_ at 37°C, then induced with a final concentration of 0.5 mM IPTG (isopropyl β-d-1-thiogalactopyranoside), while dropping the temperature to 20°C. After 5 h, cells were harvested via high-speed centrifugation and stored at –80°C.

Cells were homogenized on ice with lysis buffer (100 mM Tris Base pH 8.0, 150 mM NaCl, 1 mM TCEP). Protease inhibitors Aprotinin 1000× (0.5 mg/ml), Leupeptin 1000× (0.5 mg/ml), Pepstatin A 1000× (0.7 mg/ml), & PMSF (phenylmethylsulfonyl fluoride) 150× (25 mg/ml) were added before cell lysis. Probe sonication for cell lysis was performed at settings of 4/60 (power/output). The lysate was clarified by high-speed centrifugation at 16 000 RPM for 30 min. All purification steps were performed at 4°C. If the construct contained a Strep-II tag the supernatant was loaded onto strep resin (Strep-Tactin®XT 4Flow®, IBA). The resin was washed with lysis buffer and then eluted with Strep elution buffer (100 mM Tris Base, 150 mM NaCl, 50 mM biotin, 1 mM TCEP, pH 8.0). If the construct contained a His-Sumo tag or His tag, the supernatant was loaded onto Ni-NTA resin and was eluted with His elution buffer (100 mM Tris Base, 150 mM NaCl, 500 mM imidazole, 1 mM TCEP, pH 8.0). Fractions with CasDinG were pooled and desalted (HiPrep 26/10 Desalting, GE Healthcare) into the low salt buffer (100 mM Tris Base pH 8.0, 10 mM NaCl, 1 mM TCEP). Elutions were then run over a heparin column (HiTrap Heparin HP, GE Healthcare), washed with 47.5 mM NaCl buffer, and eluted with a high salt buffer (100 mM Tris Base pH 8.0, 500 mM NaCl, 1 mM TCEP). When present, the His-Sumo tag was removed by incubating with Tabacco Etch Virus (TEV) protease at a 50:1 (CasDinG: TEV protease Absorbance 280 ratio) at 4°C and then putting the cleaved CasDinG over Ni-NTA resin to remove the cleaved tag or uncleaved protein. Samples were spin concentrated (Corning® Spin-X® UF 50 MWCO) before further purification with size exclusion (HiLoad 26/600 Superdex 200 pg., GE Healthcare) into the high salt buffer (100 mM Tris pH 8.0, 500 mM NaCl, 1 mM TCEP). Protein samples were concentrated and stored at 4°C. Protein was assessed for purification after each step via 12% SDS-PAGE. Notably, we observed that glycerol and/or freezing of the purified protein impaired CasDinG enzymatic activities.

Protein concentration was determined by UV–Vis spectroscopy (Thermofisher UV–vis Nanodrop), using the Beer-Lambert law to correct absorbance values for extinction coefficient as determined by Expasy Protparam (18).

### Clustal omega alignments and EMBOSS NEEDLE pair-wise alignments

*E. coli* (P27296) and *S. aureus* (Q2FGY5) DinG sequences were obtained through the Uniprot database server and aligned to *P. aeruginosa 83* CasDinG using the Clustal Omega multiple sequence alignment tool (19). Pairwise sequence alignments were performed between *Pa83* CasDinG and *E. coli or S. aureus* sequences using an EMBOSS Needle alignment (20). Structural alignments between domains were either performed with the Secondary Structure Matching tool in Coot (21,22), the Dali server (23), or the align command in the PyMOL Molecular Graphics System, Version 2.0 Schrödinger, LLC.

### Nucleic acid substrate preparation

Nucleic acids were synthesized by Integrated DNA Technologies (IDT) (Supplementary Table S1). Nucleic acids were labeled with a fluorescein (FAM) label on the 5′ or 3′ end by IDT. To make duplexed nucleic acids, complementary oligonucleotides were mixed in an equimolar ratio in the presence of NEB buffer 2.1 and heated to 95°C. These oligonucleotides were slowly cooled to room temperature before being run on 12–15% NATIVE PAGE gels. Duplex bands were then gel extracted, ethanol precipitated, and reconstituted in water. For a list of oligonucleotides used in assays, refer to Supplementary Table S1.

### Malachite green ATPase assays

Concentrations of P_i_ were determined with a Malachite Green Phosphate Assay kit (BioAssay Systems, Hayward, CA, USA). Activated Malachite Green reagent was added to wells of a 384 well plate (Corning Assay Plate, 384 wells, Black with clear bottom, non-binding surface, Low flange, no lid, polystyrene, 3766). Before the reaction, assay components were pre-incubated at 37°C for 10–15 min. Reactions were started with the addition of ATP and were run at 37°C. The reaction was quenched in the activated Malachite Green reagent at time points, between 30 and 150 s. The quenched reactions were developed for 30 min before sample measurement. The absorbance values of the samples were obtained using a Synergy H4 Hybrid Multi-Mode Microplate Reader measuring absorbance at 620 nm.

Comparison of initial velocities with various nucleic acid substrates utilized 10 nM CasDinG, 100 nM nucleic acid, and 600 μM ATP that was reconstituted in buffer (50 mM Tris pH 7.5, 0.1 mg/ml Recombinant Albumin, 1 mM MgCl_2_, 1 mM TCEP). Initial velocities of CasDinG NTP hydrolysis were determined in the presence of 10 nM CasDinG, 100 nM ssDNA (40 nt.) and 600 μM nucleotide triphosphate (NEB) in the buffer (50 mM Tris pH 7.5, 0.1 mg/ml Recombinant Albumin, 1 mM MgCl_2_, 0.4 mM TCEP). Initial ATP hydrolysis velocities were determined in the presence of 10 nM CasDinG or CasDinG mutants, 100 nM ssDNA (40 nt.), and 600 μM nucleotide triphosphate (NTP) in the buffer (50 mM Tris pH 7.5, 0.1 mg/ml Recombinant Albumin, 1 mM MgCl_2_, 1 mM TCEP).

Michaelis-Menten curves were generated for CasDinG (10 nM) with single-stranded DNA (100 nM) and without (basal). Michaelis-Menten kinetics utilized the ATPase buffer (50 mM Tris pH 7.5, 0.1 mg/ml of Recombinant Albumin or Bovine Serum Albumin, 1 mM MgCl_2_, 0.4 mM TCEP). Initial velocities were performed with increasing concentrations of ATP (11–2000 μM), and a standard curve was generated using known concentrations of orthophosphate (0–100 μM). Initial velocities were calculated using linear regression. For a list of oligonucleotides used in these assays, see Supplementary Table S1. GraphPad Prism for Windows version 9.3.0 was used to fit the data using non-linear regression to the Michaelis-Menten equation (Equation 1). Where υ is the initial reaction velocity of the reaction, [*S*] is the ATP concentration, *K*_M_ is the Michaelis constant, and *V*_max_ is the maximum velocity of the enzyme.


(1) \begin{equation*}\upsilon = {{V_{\rm max}}}\left[ {{S}} \right]{\mathrm{ }}/{\mathrm{ }}\left( {{{{K}}}_{\mathrm{M}} + {\mathrm{ }}\left[ {{S}} \right]} \right)\end{equation*}

ATP concentrations were spectroscopically verified at 280 nm with crystal cuvettes and a spectrophotometer. Concentrations were confirmed using the Beer–Lambert law (Equation 2). Where *A* is the absorption, ϵ is the extinction coefficient in M^−1^ cm^−1^, *l* is the path length (cm), and *c* is concentration (M). The molar extinction coefficient used for ATP was 15 400 M^−1^ cm^−1^. Stock ATP was frozen at –80°C.


(2) \begin{equation*}{{A = }}\varepsilon {{lc}}\end{equation*}

### Helicase assays

#### Substrate comparisons and CasDinG mutant analysis

15 nM 5′ Fluorescein (FAM) labeled nucleic acid duplexes were incubated in the presence of 100 nM WT CasDinG or CasDinG mutants and 1 mM ATP in the helicase buffer (50 mM Tris pH 7.5, 1 mM MgCl_2_, 1 mM TCEP, 0.1 mg/ml recombinant albumin) for approximately 20 min at 37°C before being quenched in 2× STOP Buffer (10 mM EDTA (Ethylenediaminetetraacetic acid), 1% SDS (Sodium Dodecyl Sulfate), 20% glycerol) with 475 nM unlabeled quenching oligo (Supplementary Table S1) to prevent reannealing. Samples were run on 15% TBE Native gels.

#### NTP comparisons

15 nM 5′ FAM-labeled nucleic acid was incubated in the presence of 100 nM WT CasDinG and 1 mM ATP Analogue (ATP, ADP, ATPγS and AMP-PNP) in the helicase buffer (50 mM Tris pH 7.5, 1 mM MgCl_2_, 1 mM TCEP, 0.1 mg/ml recombinant albumin) for approximately 20 min at 37°C before being quenched in 2× STOP Buffer with 475 nM unlabeled quenching oligo. Samples were run on 15% TBE NATIVE gels

#### Metal comparisons

15 nM 5′ FAM labeled nucleic acid was incubated in the presence of 100 nM WT CasDinG and 1 mM ATP and 1 mM divalent salt (MgCl_2_, MnCl_2_, ZnCl_2_, CaCl_2_, NiCl_2_, CoCl_2_, CuCl_2_) in the helicase buffer (50 mM Tris pH 7.5, 1 mM TCEP, 0.1 mg/ml BSA) for approximately 20 min before being quenched in 2× STOP Buffer with 475 nM unlabeled quenching oligo. Samples were run on 15% TBE Native gels

#### Time courses

15 nM FAM-labeled nucleic acid substrate was exposed to 25 nM CasDinG or CasDinG mutants over 10 min in the presence of 1 mM MgCl_2_ and 1 mM ATP in the helicase buffer (50 mM Tris pH 7.5, 0.1 mg/ml recombinant albumin or bovine serum albumin (BSA), 1 mM TCEP) at 37°C. Samples were quenched in 2× STOP Buffer with 475 nM unlabeled quenching oligo, between 0 and 10 min, and run on 15% or 20% TBE Native PAGE gels. The mutant analysis utilized the same nucleic acid substrate as WT CasDinG and an equivalent amount of protein.

#### Gel analysis

All NATIVE PAGE gels for helicase assays were imaged using a BioRad Imaging system and analyzed using BioRad ImageLab software. Percent unwound were quantified using ImageLab software and the reported data is the average of three experiments, error bars when present represent the standard deviation from the mean. Graphs were made in GraphPad Prism for Windows version 9.3.0.

### Nucleic acid-binding assays

Nucleic acid-binding activities of strep-tagged WT CasDinG and CasDinG mutants were monitored using a fluorescence polarization-based assay. Anisotropy data were collected using a BioTek Synergy H4 Hybrid Multi-Mode Microplate Reader with polarizers and bandpass filters. The polarizers and bandpass filters provided 485 ± 20 nm excitation and detection of fluorescence emission at 528 ± 20 nm. Each reaction (80 μl) contained a limiting concentration (10 nM) of 3′ FAM-labeled ssDNA (40 nt). CasDinG and 3′-end FAM-labeled nucleic acid were assayed at room temperature with increasing concentrations of CasDinG (0–2.5 μM) in a binding buffer (100 mM Tris pH 8.0, 1 mM TCEP and 5 mM MgCl_2_) with or without 1 mM AMP-PNP (Sigma Aldrich). Change in anisotropy relative to FAM-nucleic acid was plotted as a function of CasDinG concentration. The apparent dissociation constant (*K*_d_) for the nucleic acid substrate was determined by fitting the raw data to a single site saturation binding model in GraphPad Prism for Windows version 9.3.0.

### Crystallization and structure determination

Strep-tagged CasDinG protein was concentrated to 5 mg/ml and crystallized using 225 mM Imidazole pH 8.0, 3.5% PEG 8000 and 4% sucrose mother liquor with hanging-drop vapor diffusion at room temperature. The crystal used for structure determination was retrieved from a drop set up with 1 μl (5 mg/ml) of protein solution to 3 μl mother liquor. The crystal was then soaked in a cryoprotectant solution composed of 30% ethylene glycol and mother liquor, mounted on a loop, and cooled to 100 K. Diffraction data were collected at the SSRL beamline 9–2. The data were indexed, integrated, and scaled using HKL3000 to 2.95 Å resolution with the space group P6_5_ (24). Phases were determined by molecular replacement in Phaser (25,26) with an N-terminally truncated Alphafold structure prediction with Colab (27), prepared with the Process Predicted Model tool in Phenix to adjust the model B-factors (28). After obtaining an initial solution with Phaser, RESOLVE was used to obtain a density-modified map (29). Because the unit cell consisted of ∼70% solvent, density modification significantly improved the electron density maps. Model building was performed in Coot (22), structures were refined using PHENIX, and validation was performed using Molprobity within PHENIX and the PDB deposition servers (28).

### Preparation of electrocompetent cells for PAM assay

50 μl of chemically competent HMS174(DE3) cells were first doubly transformed with roughly 20 ng each of plasmids #1284 (pCDF_Pa_csf1_csf2_cas6) and #1290 (pACYC_PaCR83_csf3_CasDinG) containing the PaIV-A CRISPR immune system with CasDinG (targeting). Another vial of HMS174(DE3) cells was also transformed with plasmids #1284 and #1291 (pACYC-csf3-CasDinG) which lacked a CRISPR (control). Cells were then plated with antibiotic selection by Chloramphenicol and Streptomycin and allowed to grow overnight at 37°C. The next day 3 well-isolated colonies were picked from both a targeting plate and a control plate and each colony was used to inoculate a 5 ml overnight growth of LB with selection by chloramphenicol and streptomycin. Overnight growths were carried out in a shaking incubator at 37°C.

The following day, 2 ml of each overnight growth was used to inoculate 48 ml of fresh LB with selection antibiotics, and the culture was then allowed to grow until reaching an OD of approximately 0.25. At this point, 0.1mM IPTG was added to induce expression of the CRISPR-Cas system, and the cells were allowed to grow for another 50 min. The cultures were then transferred to 50 ml conical tubes and spun down at 4000 × g at 4°C for 15 min to pellet them. Pellets were then resuspended in 50 ml of ice-cold sterile MilliQ filtered water followed by another round of centrifugation at 4000 × g at 4°C for 15 min. Pellets were then resuspended in 2 ml of ice-cold sterile 10% glycerol in a new 15 ml conical tube and spun down again at 4000 × g at 4°C for 10 min. Cells were resuspended one more time in 460 μl of ice-cold 10% glycerol and used immediately for electroporation.

### *In vivo* PAM assay

A lyophilized PAM library in the form of a pET backbone containing a CA01 target sequence flanked on the 5′ end by a library of all potential base combinations of the five positions immediately upstream of the target was provided by the Chase Biesel laboratory at the Helmholtz Center for Infection Research, and resuspended in nuclease-free water to a concentration of roughly 500 ng/μl. 1.5 μl of the PAM library was then added to 100 μl of a vial of fresh electrocompetent cells containing either a functional (targeting) or non-functional (control) immune system. This was done in biological triplicate with three targeting and three control transformations. The cells were then transferred to a pre-chilled 2 mm electroporation cuvette. Recovery media consisting of SOB with 0.1 mM IPTG was prepared for each transformation. Electroporation was carried out for each cuvette with settings of 2500 V, 25 μF, and 400 Ω, with a typical time constant of 4–5 ms. Immediately after applying the voltage 1 ml of recovery media was added, and the cells were transferred to 1.5 ml microcentrifuge tubes to recover in the incubator at 37°C with shaking.

After recovery, 1 ml of each transformation was used to inoculate 50 ml of LB with triple antibiotic selection (chloramphenicol 25 μg/ml, streptomycin, and kanamycin 50 μg/ml). Liquid cultures were then allowed to grow overnight for 20 h at 37°C with shaking, and then harvested by centrifugation at 4000 × g at 4°C for 15 min. Plasmid DNA was then extracted from each pellet with an Omega Bio Tek E.Z.N.A.® Plasmid DNA Midi kit.

### Next-generation sequencing

Primers flanking the insertion site in the plasmid were used to amplify the PAM sequences from the PAM library from each of the three targets and three control samples. To these primers were added the Illumina Truseq sequencing primer sequences. This reaction was performed for 22 cycles using Q5 (New England Biolabs). These Illumina sequences were then used as the template for a second round of PCR to add indexes and the p5 and p7 Illumina adapter sequences to make finished sequencing libraries. This PCR was performed for 10 cycles using Q5. These libraries were then sequenced on an Illumina MiSeq sequencer using the 300-cycle v2 chemistry. After demultiplexing approximately 500 000–600 000 reads were obtained for each sample.

### PAM sequence analysis

A short Python script was used to parse the five nucleotides preceding a correct target sequence from each read, and the number of occurrences of each potential NNNNN PAM was counted and exported as a CSV file. Counts were summed for all targeting and control runs, and a depletion score was calculated for each PAM sequence which relates the proportion of all counts of a PAM in the targeting runs to the control runs.


\begin{eqnarray*} && {\mathrm{Depletion Score = }}\frac{{total\ control\ reads}}{{total\ targeting\ reads}}\nonumber\\ && \quad * \frac{{control\ PAM\ reads}}{{targeting\ PAM\ reads}}\end{eqnarray*}

All PAM sequences along with their depletion scores were then input into a Krona plot generator (30,31) which was in turn used to generate a PAM wheel.

### Preparation of chemically competent type IV-A CRISPR-cas cells

HMS174(DE3) *E. coli* cells (Novagen), were transformed with 50–100 ng of both plasmid #1284(pCDF_Pa_csf1_csf2_cas6) and plasmid #1290(pACYC-PaCR83-csf3-DinG) containing WT CasDinG, or plasmids containing mutant CasDinG, (PACYC-PA83CR-csf3-DinG-ArchKO), (PACYC-PA83CR-csf3-DinG-NT-KO) (PACYC-PA83CR-csf3-DinG-FeS-KO), (PACYC-PA83CR-csf3-DinG-FeS-loop-switch), (PACYC-PA83CR-csf3-DinG-R196H), (PACYC-PA83CR-csf3-DinG-D337A_E338A) or (PACYC-PA83CR-csf3-DinG-R706W). Mutants were generated using the Q5 Mutagenesis kit (NEB Biolabs).

Colonies from the overnight plates were used to inoculate 25 ml of the prepared LB broth in a 50 ml Falcon tube. These cells were then incubated at 37°C until an OD_600_ between 0.2–0.3 when they were induced with 0.1 mM IPTG (100 μM IPTG). Cells were allowed to grow for an additional 45 min at 37°C before being cold-shocked on ice for 20 min. Cells were then spun down at 2700 × g for 15 min at 4°C. The supernatant was decanted, and the cells were resuspended in 12 ml RF1 Buffer (100 mM RbCl, 50 mM MnCl_2_·4H_2_O, 30 mM Potassium acetate, 15% m/v glycerol). Cells were then allowed to rest on ice for 15 min before being spun down at 870 x G for 15 min. The supernatant was decanted and cells were resuspended in 1 ml of RF2 Buffer (10 mM MOPS, 10 mM RbCl, 75 mM CaCl_2·_2H_2_O 15% m/v glycerol). The cell solution was incubated on ice for 15 min prior to aliquoting cells in 100 ul volumes and flash freezing at −80°C.

### *In vivo* plasmid competition assay

Chemically competent HMS174(DE3) cells containing plasmids # 1284 and 1290 were transformed with 30 ng of target or non-target plasmid, #2380 (pET27b_CA01_GGAAA) and #1095 (pET27b(+)-non-target_TTTC) respectively. Cells were then heat shocked at 42°C for 30–40 s, followed by cold shock on ice for 1–3 min. 400 μl of LB containing 0.1 mM IPTG were then added to the cold-shocked cells. Cells were then incubated at 37°C for 45–50 min in a shaking incubator, 200 RPM, followed by plating of cells onto a triple antibiotic LB agar selection plate (Chloramphenicol 25 μg/ml, streptomycin and kanamycin 50 μg/ml, and IPTG 0.1 mM IPTG). The cells were then spread around by shaking with sterile glass beads per plate and subsequently allowed to dry before being placed in a 37°C incubator for 24 h. Colonies were then counted manually for analysis.

### Circular dichroism

CD spectra were collected for WT strep-tagged DinG and CasDinG mutants at an approximate concentration of 0.5 mg/ml. Protein solutions were diluted into the low salt buffer (100 mM Tris Base pH 8.0, 10 mM NaCl, 1 mM TCEP) prior to data collection. Data were collected using a JASCO model J-1500 spectropolarimeter. CD spectra were collected from 190 to 260 nm at 10°C using 0.1 cm quartz cuvettes, 1 nm data sampling, a 50 nm/min scan rate, and a 2-second data integration time. Measurements were converted from machine units to Δϵ units by using the equation Δϵ = θ × (0.1 × MRW)/ (*P* × Conc) × 3298, where MRW is mean residue weight, *P* is pathlength, and Conc is protein concentration, as suggested by Dichroweb (32).

### Structure prediction and comparison

Alphafold2 Colab was used to predict the atomic structure of the N-terminal domain of CasDinG (27). The Dali server and Foldseek were used to identify structurally homologous structures in the PDB (Dali) and Alphafold (Foldseek) databases (23,33). PyMoL was used to generate comparative figures.

## RESULTS

### CasDinG is a DNA-dependent ATPase

It was recently demonstrated that mutation of the CasDinG Walker A (EAATGTGKG amino acids 138–146) or Walker B (VDEAHLL amino acids 336–342) motif disrupts type IV-A immunity (7,8). Although the canonical function of these motifs is ATP binding and hydrolysis, it remained unclear if CasDinG uses ATP to unwind DNA duplexes, like *E. coli* DinG (13), or to regulate an accessory activity, like *S. aureus* DinG (16). Thus, to determine the role of ATP in CasDinG function we expressed and purified recombinant CasDinG from *P. aeruginosa strain 83* extrachromosomal plasmid (Supplementary Figure S2), and used a malachite green assay to detect the release of inorganic phosphate from ATP (Figure 1B). Because ATPase activities of helicases are often enhanced by the presence of nucleic acid (34,35), we examined ATP hydrolysis in the presence of various DNA substrates (Figure 1B and Supplementary Table S1). We observed that a 17 nt DNA duplex with a 16 nt poly-T 5′-overhang strongly enhanced ATPase activity, while a similar DNA duplex with a 3′-overhang only moderately enhanced ATPase activity, and a 17 nt blunt end duplex did not enhance ATPase activity above background. These data suggested that CasDinG ATPase activity is enhanced by nucleic acid, with a preference for substrates with 5′ single-stranded (ss)DNA overhangs.

To determine how nucleic acid length influences CasDinG ATPase activity, we examined activity in the presence of a 17 and 40 nt random sequence ssDNA. The 40 nt ssDNA substrate enhanced activity similar to the 5′-overhang duplex substrate, while the 17 nt ssDNA substrate did not enhance ATPase activity above the background (Figure 1B). Interestingly, a 40 nt ssRNA substrate with the same sequence, but phosphorothioated at approximately every 5th position (Supplementary Table S1) and a 17 nt RNA did not enhance ATPase activities, suggesting the ATPase activity of CasDinG is preferentially enhanced by DNA.

To evaluate whether CasDinG prefers to hydrolyze a specific ribonucleotide triphosphate we compared the release of inorganic phosphate from ATP, GTP, CTP, and UTP (Figure 1C) in the presence of a 40 nt ssDNA. CasDinG hydrolyzed GTP and UTP, but at a rate fourfold lower than ATP, and CTP was not hydrolyzed, indicating CasDinG preferentially hydrolyzes ATP.

To further evaluate how nucleic acid enhances CasDinG ATPase activity, we collected ATP hydrolysis velocities with or without a constant concentration of 40 nt ssDNA, and at different concentrations of ATP. Velocity values were then fit to the Michaelis Menten equation (Figure 1D and Supplementary Figure S3). Without ssDNA CasDinG only weakly hydrolyzed high concentrations of ATP, disallowing a fit to the Michaelis Menten equation. In contrast, the 40 nt. long ssDNA enhanced CasDinG activity to a *k*_cat_ of 32 ± 1 s^−1^ (Supplementary Figure S3), similar to the observed ATPase activity of *E. coli* DinG in the presence of ssDNA (*k*_cat_ of 24 s^−1^)(13).

Just as the presence of nucleic acid can enhance the ATPase activity of helicases, helicase affinities for nucleic acid substrates are sometimes enhanced by the presence of ATP (36–38). To investigate if ATP enhances the ability of CasDinG to bind nucleic acid substrates we determined the dissociation constant (*K*_d_) of binding of our 40 nt. long ssDNA substrate to CasDinG with and without the non-hydrolyzable analog AMP-PNP (Figure 1E). CasDinG bound to the ssDNA with a *K*_d_ of 25 ± 3.1 nM without AMP-PNP, and with a *K*_d_ of 26 ± 2.9 nM with AMP-PNP, demonstrating the presence of a nucleotide triphosphate does not enhance nucleic acid binding. Collectively, these data indicate CasDinG is an ATP hydrolase that is activated by ssDNA. However, the general affinity of CasDinG for nucleic acids is not influenced by the presence of nucleotides.

### CasDinG is an ATP-dependent 5′-3′ DNA translocase that unwinds DNA/DNA and RNA/DNA duplexes

**Figure 2.**
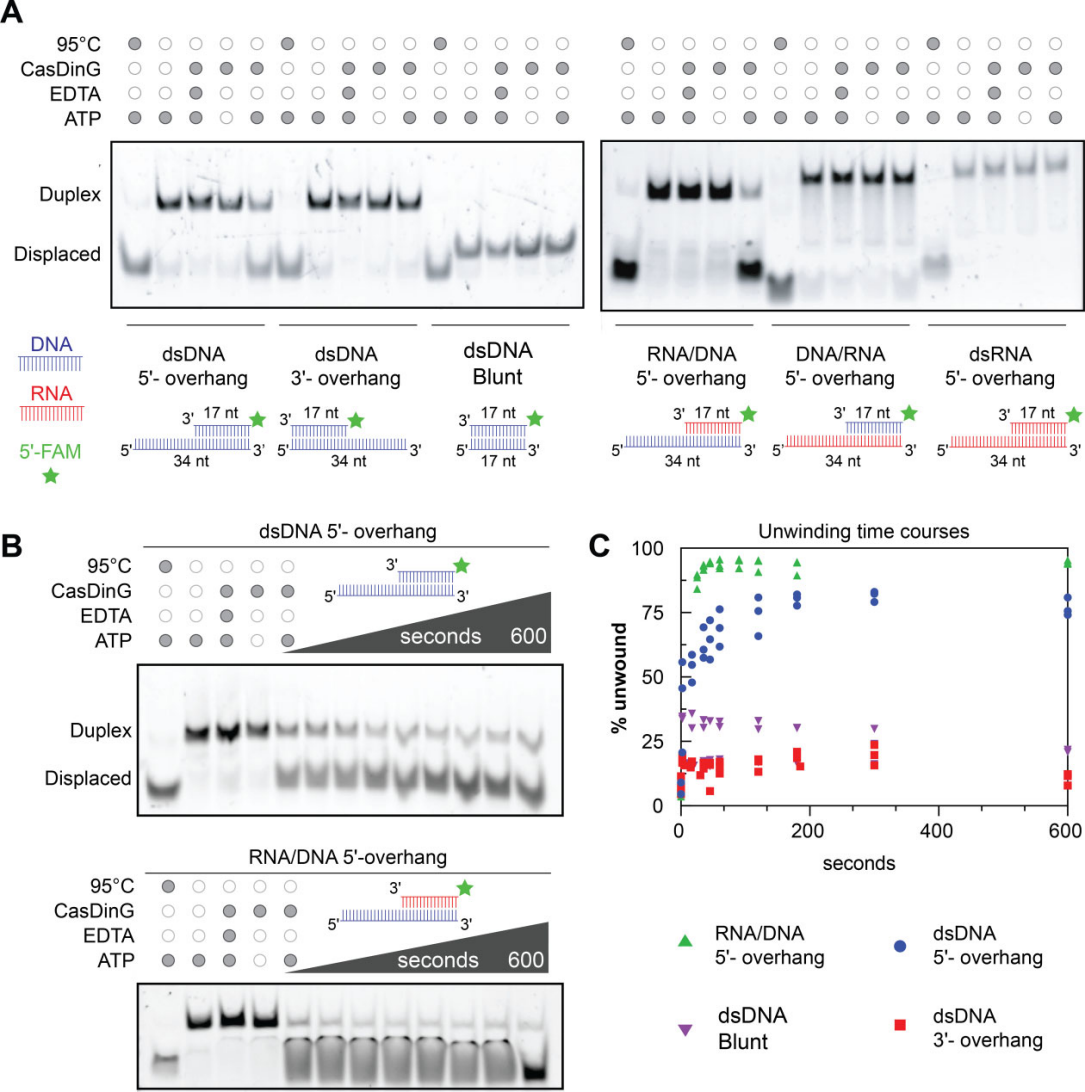
CasDinG is a 5′-3′ ATP and metal-dependent helicase. (**A**) Native PAGE helicase assays showing displacement of a FAM-labeled ssDNA or ssRNA from various nucleic acid duplexes. Minimal to no displacement is visualized for 3′ overhangs, blunt-ended duplexes or substrates in which the 5′ overhang is RNA. All reactions were quenched after 20 min. (**B**) Native PAGE helicase assays showing time course unwinding of 5′-FAM labeled dsDNA 5′-overhang and 5'-FAM labeled RNA/DNA 5'-overhang. The reactions took place for 10 min using 10 nM CasDinG and 15 nM FAM labeled duplexed substrates. (**C**) A graph plotting the % of displaced FAM-labeled nucleic acids over time with various duplexes. Only the dsDNA 5′-overhang and RNA/DNA hybrid 5′-DNA overhang unwound >50%.

It remained unclear if CasDinG ATPase activity was coupled to nucleic acid translocation and duplex unwinding. To determine if CasDinG possesses helicase activity we combined CasDinG with various DNA duplexes in the presence of ATP and Mg^2+^. One strand of each duplex was 5′-end-labeled with fluorescein (FAM) to visualize strand displacement with an electrophoretic mobility shift assay (EMSA) on a polyacrylamide gel. We observed that CasDinG displaced the FAM-labeled strand from DNA duplexes with 5′-overhangs, but not duplexes with a 3′-overhang or blunt ends, consistent with a 5′-3′ unwinding polarity (Figure 2A). Additionally, CasDinG displaced RNA from RNA/DNA hybrid duplexes with 5′-DNA overhangs, but CasDinG did not displace DNA or RNA from duplexes with 5′-RNA overhangs, consistent with a preference for translocation on ssDNA (Figure 2B).

The unwinding activities of CasDinG were only observed in the presence of ATP and were impaired by EDTA, indicating unwinding is coupled to ATP hydrolysis and the presence of a divalent metal. To confirm ATP hydrolysis is coupled to unwinding we examined helicase activity in the presence of ADP and non-hydrolyzable analogs ATPγS and AMP-PNP. Only in the presence of ATP was duplex unwinding observed above 20% (Supplementary Figure S3A and B). To determine which divalent metals activate CasDinG helicase activity, we examined helicase activity with a variety of divalent metals. We observed that Mg^2+^, Mn^2+^, Ca^2+^, Ni^2+^ and Co^2+^, allow for helicase activity, whereas Zn^2+^ and Cu^2+^ did not (Supplementary Figure S3C). Collectively these data demonstrate that CasDinG is an ATP- and divalent metal ion-dependent helicase that preferentially translocates in the 5′-3′ direction on ssDNA strands and displaces DNA or RNA complements.

### CasDinG structure consists of a helicase core with three accessory domains

**Figure 3.**
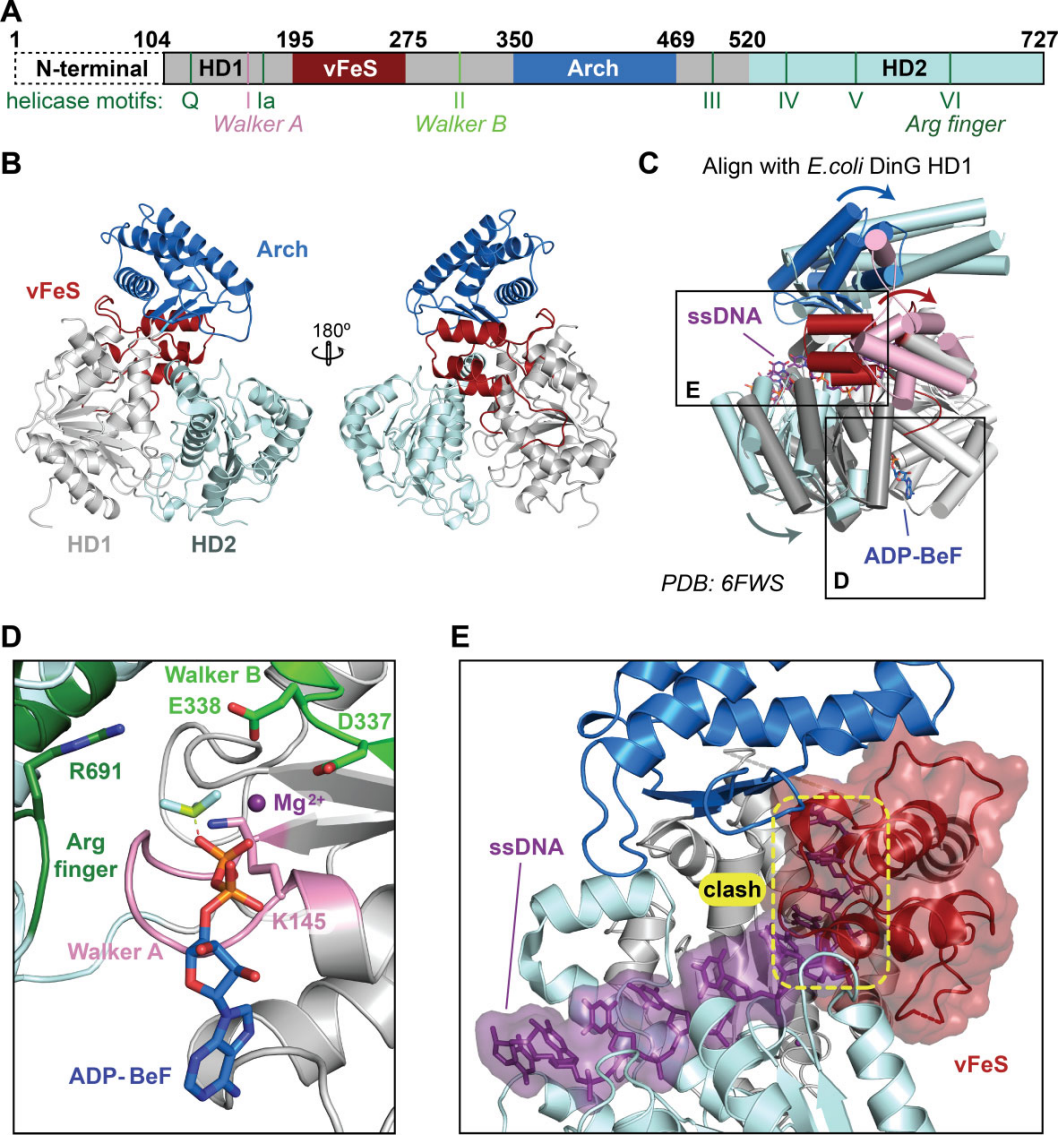
X-ray crystal structure of CasDinG. (**A**) Diagram of the CasDinG protein sequence depicting the HD1 (silver) and HD2 domains (pale cyan) with accessory domains vFeS (red) and Arch (blue). The positions of SF2 helicase motifs are indicated under the diagram. The N-terminal domain of CasDinG is indicated but was unresolved in the solved structure. (**B**) Model of CasDinG highlighting the Arch and vFeS accessory domains. (**C**) Alignment of the CasDinG model with the *E. coli* DinG model bound to ssDNA and ADP-BeF3 Mg^2+^ complex (PDB 6FWS). Positional differences between the CasDinG and *E. coli* DinG are indicated with arrows. (**D**) Zoomed in view of the ATP binding pocket with the aligned ADP-BeF3 Mg^2+^ complex (6FWS) to the CasDinG model. The Walker A (pink) and Walker B (lime) motifs are in a position to coordinate the ADP-BeF3 Mg^2+^. (**E**) View of the nucleic acid binding pocket showing the aligned ssDNA (6FWS) within the RecA-like fold of the HD1 and HD2 domains. Highlighted in yellow is a clash between the aligned ssDNA and the CasDinG vFeS domain, indicating the vFeS domain is in a closed position that would have to open to accommodate bound ssDNA.

CasDinG shares less than 22% sequence identity and 31% sequence similarity with *S. aureus* and *E. coli* DinG (Supplementary Figure S1). The highest similarity regions are the SF2 helicase motifs (Q, I, II, III, IV, V, VI) within the two RecA-like domains of the helicase core, while the least similar regions reside within the predicted accessory domains (Supplementary Figure S1). We hypothesized that amino acid differences in the accessory domains might influence the function of CasDinG in type IV-A immunity. To better understand the function of the accessory domains we determined the crystal structure of CasDinG from *P. aeruginosa strain 83* at 2.95 Å resolution with an Rwork/Rfree of (18.5 / 21.5) in space group P 6_5_ (Figure 3 and Table 1). We solved the structure by molecular replacement using an AlphaFold2 model of N-terminally truncated CasDinG (25–27). Notably, the domains of the AlphaFold2 prediction aligned to our final model with RMSDs <1 Å (Supplementary Figure S4). However, the positioning of the accessory domains was slightly different between the AlphaFold2 model and our final structure.

**Table 1.** Data collection and refinement statistics

Dataset	Native
**Data collection**	
Beamline	SSRL 9–2
Space group	*P*6_5_
Cell dimensions	
*a*, *b*, *c* (Å)	123.3, 123.3, 136.9
α, β, γ (deg)	90.0, 90.0, 120
Wavelength (Å)	0.88684
Resolution^†^ (Å)	50–2.95 (3.06–2.95)*
*R*_merge_(%)	18.8 (135.6)
CC_1/2_	0.994 (0.766)
*I* / σI	20.0 (2.0)
Observations	49009 (4849)
Unique reflections	24916 (2453)
Multiplicity	20.5 (16)
Completeness (%)	100.0 (99.8)
Resolution (Å)	50–2.95 (3.06–2.95)
No. reflections	24916
**Refinement** *R*_work_/*R*_free_ (%)	18.5/21.5
No. atoms	
Protein	4578
Water	0
Ligands	0
*B*-factors	
mean	76.2
R.m.s. deviations	
Bond lengths (Å)	0.002
Bond angles (deg)	0.52
Ramachandran	
Favored (%)	96.33
Allowed (%)	3.77
Outliers (%)	0
Clashscore	3.4

*Values in parentheses are for highest-resolution shell.

^†^Resolution limit used the criterion of I/σI > 2.0

The CasDinG structure reveals a SF2 helicase core of two RecA-like helicase domains (HD1 and HD2) and two accessory domains inserted within HD1 (vFe/S and arch domain) (Figure 3A). An N-terminal domain of 103 amino acids was not observed in the electron density. Similar to other SF2 helicases, the conserved helicase motifs decorate the cleft between HD1 and HD2 (37) (Supplementary Figure S5). Alignment of the RecA helicase domains of CasDinG with *E. coli* DinG bound to ADP-BeF and ssDNA (PDB: 6FWS) reveals high similarity within the helicase core and suggests CasDinG relies on the conserved helicase motifs to bind and hydrolyze ATP with a mechanism similar to *E. coli* DinG (Figure 3C and D) (15). Additionally, several residues linked to XPD disease states that lie outside the conserved helicase motifs (e.g. R196, R614, and R706) are structurally conserved (Supplementary Figure S1 and Supplementary Figure S6)(39).

Recent structures of *E. coli* DinG bound to ssDNA were determined in the absence (PDB:6FWR ‘open conformation’) and presence (PDB:6FWS ‘closed conformation’) of an ATP analog, revealing two distinct conformations that suggest a two-step mechanism for DinG-mediated DNA translocation and duplex unwinding (Supplementary Figure S7) (15). In the first step, ATP binding causes HD2 to slide along the ssDNA in the 5′-3′ direction towards HD1, while the ssDNA is held in place by HD1. The increased proximity of the RecA domains brings the two halves of the ATPase active site together, promoting ATP hydrolysis. In the second step, ATP hydrolysis and ADP release switch the affinities of the HD domains for ssDNA, causing HD2 to hold to the 5′ side of ssDNA while HD1 translocates along the ssDNA in the 5′-3′ direction. As the RecA domains translocate, it is proposed that the accessory Arch and FeS domains would sterically displace complementary strands (Supplementary Figure S7) (15).

To determine what our CasDinG structure could reveal about the unwinding mechanism of DinG-like helicases, we aligned HD1 of CasDinG with HD1 of the *E. coli* DinG binary structure or the ‘open’ conformation (rmsd of 3.5 Å) (Supplementary Figure S7C). The alignment revealed that the HD domains of CasDinG are in a wider, or ‘extra open’, conformation than the HD domains of the binary *E. coli* DinG structure (Supplementary Figure S7D). The extra open conformation may be a crystallographic artifact, or the result of having no ssDNA bound in the CasDinG structure. The second possibility implies ssDNA binding would bring HD1 and HD2 closer together to adopt a conformation like that observed in the binary *E. coli* DinG structure.

Similar to DinG and XPD-family helicases, CasDinG contains two accessory domains inserted within HD1. Although the two inserts in CasDinG have low sequence similarity to *E. coli* DinG, they share tertiary topology (Supplementary Figure S8). The first insert (residues 195–275) consists of a four alpha-helix bundle that aligns with the *E. coli* DinG FeS cluster domain with an RMSD of 2.3 Å using the Coot Secondary Structure Matching tool (15,21,22). Despite this conserved topology, CasDinG lacks three of the four cysteines observed in *E. coli* DinG that coordinate the FeS cluster, and no FeS cluster is observed in the electron density of the CasDinG structure (Supplementary Figures S1 and S8A). The FeS cluster coordination in *E. coli* DinG stabilizes the tertiary fold of the domain, holding α2 in the proximity of the loop directly downstream of α4 (Supplementary Figure S8A). In CasDinG, the lack of a stabilizing FeS cluster is compensated for by a salt bridge formed between residues R204 and D269 (Supplementary Figure S1). These salt bridge residues appear to be fairly conserved in CasDinG sequences but not in chromosomal DinG (17). Thus, to distinguish the CasDinG domain from DinG accessory domains that coordinate FeS clusters, we named this domain in CasDinG a vestigial FeS domain or vFeS. Additional differences between CasDinG and *E. coli* DinG in this domain include the lack of a loop-helix-loop in CasDinG that connects helices α2 and α3 of the *E. coli* DinG FeS domain, and an extended loop helix connecting alpha helices α3 and α4 in CasDinG not observed in *E. coli* DinG (Supplementary Figure S8). This extended loop sits within the cleft of the RecA domains directly above the ATP binding site suggesting it may influence ATPase or unwinding activity.

The position of the CasDinG vFeS domain is dramatically different from the position of the FeS domain in the *E. coli* DinG structures (Figure 3 and Supplemental Figure S8). When the HD1 domains are aligned, the CasDinG vFeS domain is rotated 55° and translated 23 Å away from the position of the FeS domain in the *E. coli* DinG structures (Supplementary Figure S8C). Furthermore, the vFeS domain is located in the region of the *E. coli* DinG structure that binds ssDNA (Figure 3). These differences indicate the vFeS domain would have to undergo a major conformational change from the position observed in our crystal structure to allow for ssDNA binding (Figure 3E and Supplementary Figure S8). Notably, analysis of CasDinG crystal packing suggests that the vFeS position that sterically blocks ssDNA binding is likely a crystallographic artifact, indicating a conformational rearrangement of the domain that allows for ssDNA would likely be allowed in solution.

In DinG, XPD, and CasDinG, the FeS domain interfaces with the other HD1 insert called the ‘arch’ domain (34,39–41). In the *E. coli* DinG structures the contact between the FeS and arch domains creates a pore that ssDNA passes through to interact with HD1 and HD2. While the pore created by the vFeS and arch domains in the CasDinG structure is not large enough to allow ssDNA to pass through, the PISA server (42) calculates the interface between the CasDinG vFeS and arch domains to be approximately 720 Å^2^, larger than the FeS/arch interfaces of *E. coli* DinG (238 Å^2^) and Human XPD (346 Å^2^), and indicating a similar interface between vFeS and the arch domain would likely remain, even in a different conformation that forms a pore through which ssDNA can pass during unwinding.

The CasDinG arch domain (residues 352–467) consists of a four-helix-bundle and a three-stranded anti-parallel beta-sheet, sharing structural topology with the arch domain of *E. coli* DinG and XPD helicases (15,40,41). Indeed, the Dali server reports an RMSD of 3.3 Å between aligned CasDinG and *E. coli* DinG arch domains (Supplementary Figure S9) (23). However, the arch domain of *E. coli* DinG is 61 amino acids longer than the CasDinG arch and contains an extra beta-loop connecting helices α2 and α3 (Supplementary Figure S9A). When aligned along HD1 the *E. coli* DinG arch domain appears to be ∼12 Å longer than the CasDinG arch and is rotated 25° away from the ssDNA binding site (Supplementary Figure S9C). The arch domain of XPD makes important contacts with other proteins in the human transcription factor IIH complex (40,43). Thus, it is possible that the sequence, position, and size differences of the arch domains of CasDinG and *E. coli* DinG could be associated with differences in protein-protein interactions.

### The type IV-A system recognizes a 5′-GNAWN-3′ PAM

To better understand the function of CasDinG we desired to examine structure-guided mutants with the cell-based assay we previously developed to demonstrate type IV-A plasmid clearance (7). However, we were concerned that our assay was not optimal because recent literature suggested type IV-A systems prefer a 5′-AAG-3′ protospacer adjacent motif (PAM) located on the 5′-side of the target sequence (4,8), instead of the 5′-CTTTC-3′ PAM our previous assay utilized. PAMs are small nucleotide motifs that distinguish ‘non-self’ from ‘self’ targets that reside next to the nucleic acid complements of crRNA-guided surveillance complexes (44–47).

**Figure 4.**
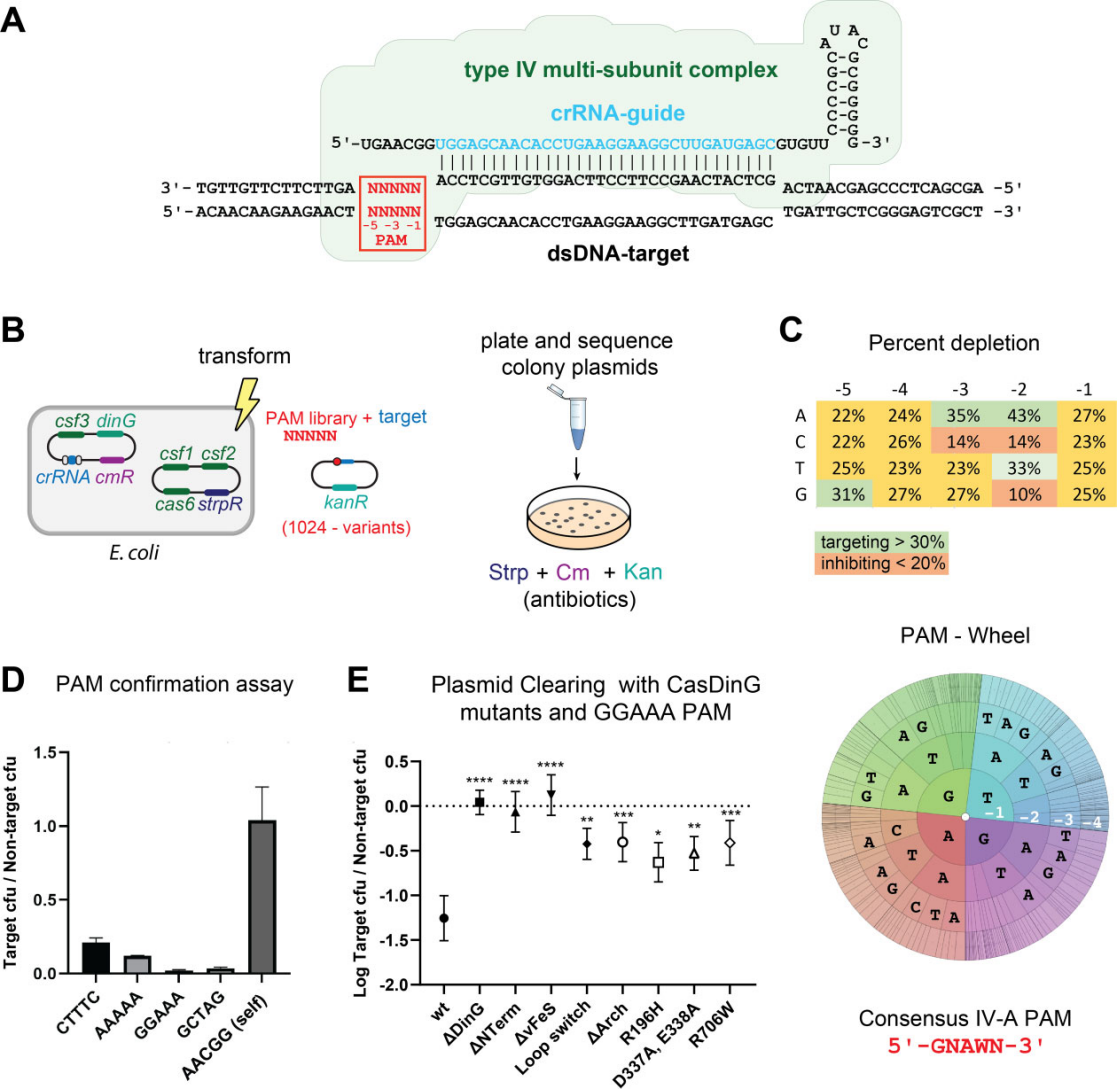
The type IV-A CRISPR system prefers a GNAWN PAM and requires CasDinG accessory domains. (**A**) Schematic depicting the type IV-A Csf complex binding to a dsDNA target. The location of the PAM is indicated in red. (**B**) Diagram of how the PAM library was performed. *E. coli* cells expressing the type IV-A system were transformed with a library of target plasmids adjacent to all possible combinations of PAM sequences. The transformation was grown under antibiotic selection, cells were harvested, and DNA was isolated and deep sequenced. (**C**) Table (top) and PAM wheel (bottom) showing percent depletion of nucleotides in the PAM assay, highlighting a consensus PAM 5′- GNAWN -3′ sequence. (**D**) The ratio of target /non-target colony forming units using four different targeting PAM sequences compared to self. (**E**) Plasmid curing assay with domain and point mutants, demonstrating these domains are essential for type IV-A CRISPR immunity.

To determine what PAM sequences optimally activate the type IV-A system, we performed a plasmid curing assay in biological triplicate with a library containing all 1024 combinations of 5 nucleotides adjacent to the 5′-side of the target sequence, similar to previous studies identifying PAM preferences (30) (Figure 4). Transfected cells were grown in liquid culture under immune system-inducing conditions, harvested, and deep sequenced. The depletion of a PAM sequence, when compared to no-immune system control, indicated an activating PAM sequence. Depletion scores for each PAM base position, and a PAM wheel (Figure 4C), were generated to reveal PAM nucleotide preferences. The data revealed a depletion preference for guanine at position −5, adenosine at position −3, and adenosine or thymine at position -2. There appeared to be no preference for nucleotides at positions −1 and −4, defining a preferred consensus PAM of 5′-GNAWN-3′, where W indicates A or T. Notably, there also appears to be an anti-targeting effect when guanine or cytosine is located at position −2, and when cytosine is located at position −3. The type IV-A CRISPR repeat adjacent to the 5′-side of the spacer (self-sequence) contains a cytosine at the −3 position and guanine at the −2 position, suggesting the PAM recognition mechanism has evolved to avoid self targets while gaining a mechanism to interrogate non-self targets.

To confirm that the 5′-GNAWN-3′ PAM is preferred we performed plasmid clearance assays with single target plasmids containing specific activating PAMs identified in the assay (Figure 4D). We first tested the 5′- CTTTC -3′ PAM used in our previous work. Although this PAM does not contain a preferred adenosine at the −3 position or a guanine at the −5 position we still observed measurable depletion of the target strand compared to the non-target. However, consistent with our PAM library screen, PAMs that conformed to the 5′-GNAWN-3′ consensus sequence, with either a −3 adenosine, a −5 guanine, or both (e.g. 5′-GGAAA-3′) showed the strongest target clearance. Collectively, these data suggest that the reason the 5′-CTTTC-3′ PAM worked previously is because it did not contain anti-targeting bases G-C or C in the −2 or −3, position. However, other PAMs that conform to the 5′-GNAWN-3′ sequence are preferred.

### CasDinG accessory domains are essential for type IV-A immunity

After identifying the optimal PAM for the type IV-A system, we used our cell-based assay to investigate the role of the CasDinG accessory domains in type IV immunity. Using our structure as a guide, we mutated the plasmid encoding CasDinG to remove regions encoding the N-terminal, vFeS, or arch domain (Supplementary Figure S10). Additionally, to compare CasDinG function to other DinG-like proteins, we replaced the loop of the vFeS cluster positioned in the RecA domain cleft with the sequence observed in the FeS of DinG, we mutated Walker B residues involved in ATP hydrolysis (D337A, and E338A), and mutated two residues outside the conserved helicase motifs known to cause XPD-related disease states (R196H and R706W) (39). We then examined how these mutant CasDinG proteins cleared a target plasmid with a 5′-GGAAA-3′ PAM in a type IV-A immune system assay.

Previous work showed mutations of respective Walker B and R706 amino acids in XPD helicases disrupted both ATPase and helicase activities, while the R196H mutation only disrupted helicase activity (39). As expected, mutation of the Walker B motif (D337A, E338A), and mutations that cause XPD-related disease states (R196H and R706W) diminished type IV-A immunity (Figure 4E), suggesting ATP-mediated helicase activities are essential to CasDinG’s role in type IV-A immunity. In addition to these disruptive point mutants, deletion of the N-terminus, the vFeS domain, and the arch domain all impaired immune system function as did mutation of the vFeS loop (Figure 4E), indicating each of these features plays an essential role in type IV-A immunity.

To better understand why domain deletions impaired type IV activity, we recombinantly expressed and purified domain deletion CasDinG proteins. While N-terminal and arch domain deletion mutants expressed and purified at wild-type levels, the vFeS deletion and vFeS loop mutant expressed poorly and could not be purified at sufficient quantities for downstream biochemical analysis. Low expression of these proteins could explain the impaired immunity phenotype observed with these mutants. To determine the stability of the mutants we were able to express and purify at wild-type levels, we performed circular dichroism to identify any major changes in the secondary structure of mutants compared to wild-type. Our results indicated similar levels of secondary structure elements for the mutants as wild-type (Supplementary Figure S11), indicating the mutations did not disrupt overall protein folding or stability.

**Figure 5.**
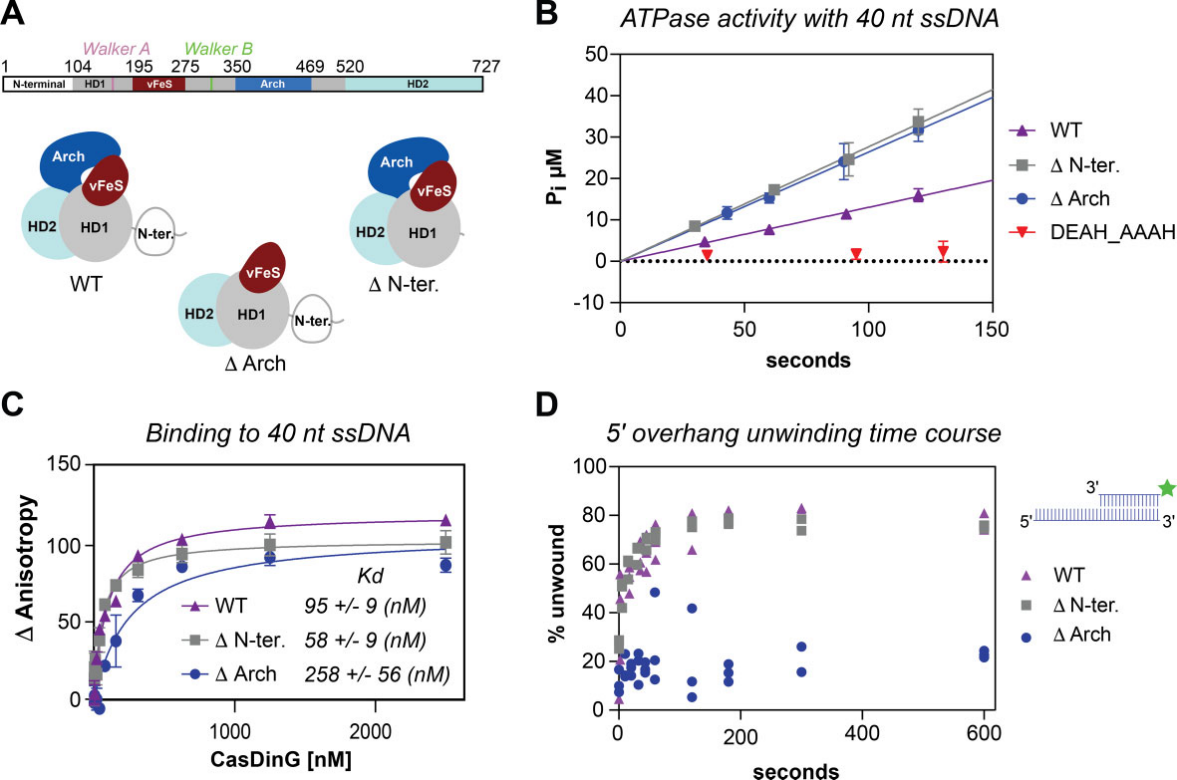
Biochemical analysis of CasDinG domain deletion mutants. (**A**) Schematic of the amino acid sequence of CasDinG (top). Cartoon depictions of WT and domain deletion proteins are drawn schematically (bottom). (**B**) The ATPase activity of WT, domain deletion proteins, and the Walker B motif mutant under ssDNA-saturated conditions. (**C**) Nucleic acid binding assay with WT, N-terminal deletion, and arch deletion proteins. (**D**) Helicase assay time course of WT and domain deletion mutants.

We next explored how the loss of the arch and N-terminal domains influenced CasDinG enzymatic activities with the same assays we used to characterize wild-type CasDinG ATPase, nucleic acid binding, and DNA unwinding activities. (Figure 5 and Supplementary Figure S12). Removal of the Arch domain impaired ssDNA binding, and disrupted helicase activity, but did not impair ATP hydrolysis activity at ssDNA saturating conditions, consistent with a role in DNA binding and unwinding nucleic acid duplexes. In contrast, the removal of the N-terminal domain did not impair the ATPase, ssDNA binding, or helicase activities, although removal of this domain impaired *in vivo* immunity. Thus, the essential role of the N-terminal domain must lie outside the canonical XPD/DinG helicase mechanisms.

### The N-terminal domain is predicted to adopt a dsDNA binding fold

**Figure 6.**
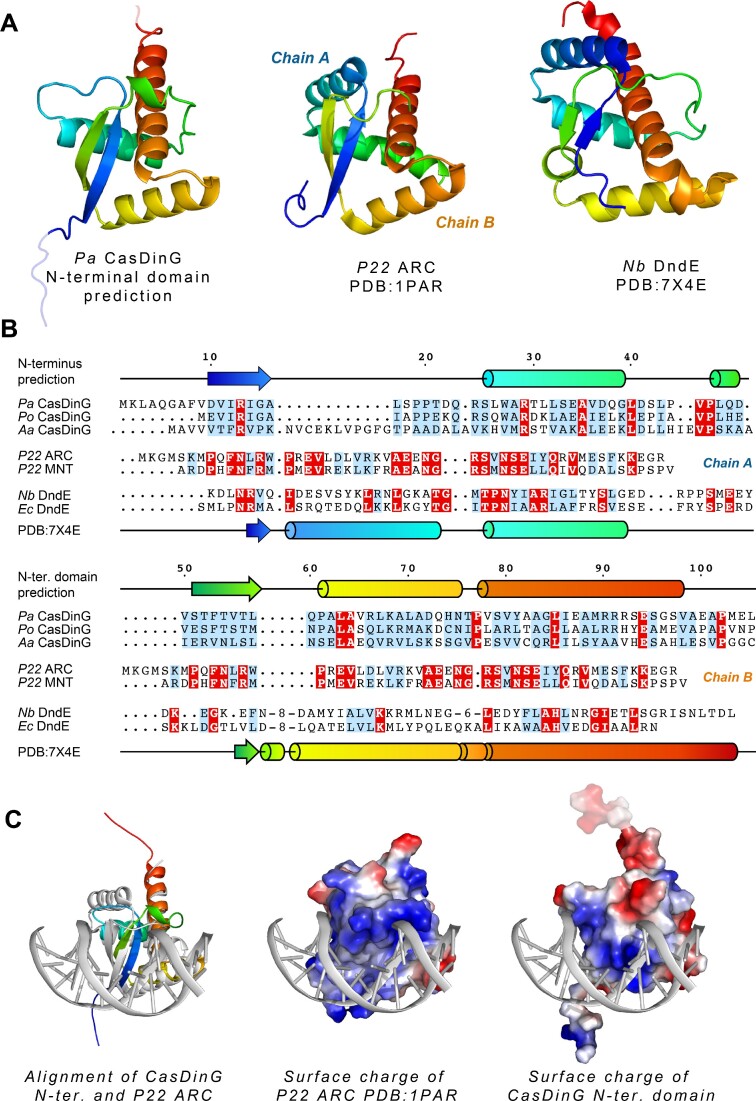
The predicted structure of the N-terminal domain reveals a putative dsDNA binding fold. (**A**) The predicted fold of the N-terminal domain (left) colored rainbow from the N- to C-terminus, positioned next to Dali search hits of the *P22* ARC repressor (middle) and the DndE protein from *Natronorubrum bangense* JCM10635 (right). (**B**) Amino acid sequence alignments of CasDinG N-terminal domains from *Pseudomonas aeruginosa* (*Pa*), *Pseudomonas oleovorans* (*Po*), and Aromatoleum aromaticum (*Aa*) (top), bacteriophage *P22* ARC and MNT repressors (middle), and DndE proteins from *Natronorubrum bangense* (*Nb*), and *Eschericia coli* (*Ec*) (bottom). Identically conserved residues are highlighted in red and similarly conserved residues are highlighted in blue. The predicted secondary structure of CasDinG is drawn schematically on top of the alignments and the secondary structure of the DndE protein is depicted at the bottom. Both proteins of the *P22* homodimer repressors are indicated to show how each monomer fits into the overall fold. (**C**) Alignment of the N-terminal domain of CasDinG with the ARC repressor bound to dsDNA (left). Surface charge depiction of the ARC repressor bound to DNA (middle). Surface charge depiction of the aligned CasDinG N-terminal domain and dsDNA.

To better understand the function of the CasDinG N-terminal domain we predicted its molecular structure with the AlphaFold2 program on the ColabFold server (27). The program predicted a compact globular fold containing three α-helices and two anti-parallel β-strands arranged in a knot-like configuration (Figure 6). This same protein fold was predicted for several CasDinG homologs, including CasDinG from other type IV-A systems that have been characterized *in vivo* or *in vitro* (e.g. *Pseudomonas oleovorans* and *Aromatoleum aromaticum*) (6,8). However, CasDinG proteins from *Klebsiella pneumonia* type IV-A systems found on lncHI1B/lncFIB plasmids appear to lack an equivalent N-terminal domain (48).

A Dali search with the predicted N-terminal domain structure revealed similarities to several nucleic acid binding proteins including bacteriophage encoded transcription regulators (49–51), the dsDNA binding VirC protein involved in DNA transfer into plants from *Agrobacterium* (52), and bacterial defense proteins such as DndE from the DNA phosphorothioate defense system (53,54) and WYL1 of the CRISPR VI-D system (55). The CasDinG N-terminal domain aligned with highest *Z* scores to several structures of a *P22* phage transcriptional regulator called the ARC repressor. Natively, the repressor is a homodimer of two ribbon-helix-helix peptides, but the highest similarity score (*Z*-score 6.6) was to an engineered version of the ARC repressor that linked the two peptides together into a single chain (50). DndE and WYL proteins adopt a similar fold as the engineered linked P22 repressor. This arrangement tucks the N-terminus of the peptide underneath a loop, forming a knot-like fold observed in another protein with structural homology to the N-terminal domain with an unknown function (56) (Figure 6A and B).

Although the predicted CasDinG N-terminal domain shares tertiary homology with several dsDNA binding proteins, their amino acid sequences are not well conserved beyond the common placement of positively charged residues on one side of the domain (Figure 6B). A structure of the ARC repressor bound to dsDNA shows that these positively charged residues interact with the backbone of DNA (Figure 6C). Alignment of the predicted N-terminal domain with the ARC repressor bound to DNA reveals the predicted N-terminal domain has a positively charged track of amino acids that could interact directly with the major groove of dsDNA in a similar fashion to the *P22* ARC repressor, suggesting the N-terminal domain may interact with dsDNA (Figure 6C).

## DISCUSSION

**Figure 7.**
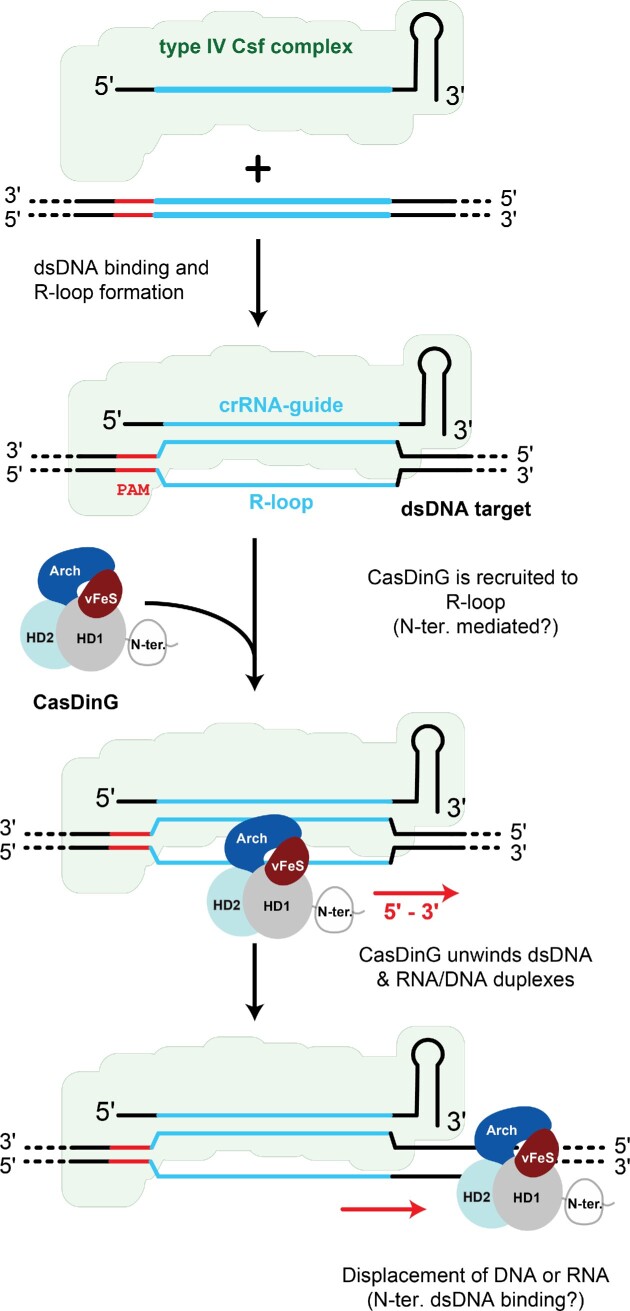
Model of the role of CasDinG in type IV-A CRISPR immunity. The type IV-A multi-subunit Csf crRNA-guided complex binds complementary dsDNA forming an R-loop to which CasDinG is recruited. CasDinG unwinds the dsDNA in a 5′-3′ manner, possibly distorting DNA structure and/or displacing RNA primers. The N-terminal domain of CasDinG likely interacts with dsDNA, but the exact role of the domain in type IV-A immunity remains unknown.

CasDinG is an essential component of the type IV-A CRISPR Cas system, which clears bacteria of invasive nucleic acid and silences gene expression (7,8). The type IV-A multi-subunit complex or Csf complex is presumed to bind to DNA targets, similar to the type I Cascade complex, and then recruit the CasDinG helicase onto the resulting R-loop (6–8) (Figure 7A). This presumption is supported by (i) recent work in *Pseudomonas oleovorans* demonstrating type IV-A systems silence LacZ expression when targeting the gene on either the coding or non-coding strand (8), and (ii) work presented here demonstrating CasDinG is a DNA, but not an RNA, translocase with dsDNA and RNA/DNA hybrid helicase activity.

Previous bioinformatic and cell-based assays suggested the Csf complex prefers to target sequences downstream of a 5′-AAG-3′ PAM (4,8). Here, we used a target library to further define the PAM preference for DNA targeting as a 5′-GNAWN-3′ PAM with a strong resistance to targeting self sequences containing a cytosine in the -3 position or guanine or cytosine in the −2 position of the PAM. Notably, the previously reported 5′-AAG-3′ PAM conforms to this broader description. Still, some individually tested PAMs in the *P. oleovorans* system that fit our proposed consensus sequence were shown to be non-targeting (8). This observation could be due to unfavored bases in the −5 position that were not considered, or species-specific differences in the type IV-A PAM recognition mechanism. It should also be noted that our assay only looked as far as the −5 position. It remains possible that other positions further downstream contribute to the self-vs-non-self PAM recognition mechanism.

Different class 1 multi-subunit CRISPR systems use distinct PAM recognition mechanisms to distinguish self from non-self targets (45). The type III systems, which target ssRNA, predominantly use a self-exclusion mechanism that inactivates interference mechanisms when the target RNA base pairs with the repeat region of the crRNA-guide (57). Alternatively, Type I systems use a protein-mediated mechanism to bind specific dsDNA PAM sequences (47). Here, we observe that the type IV-A PAM targeting mechanism is biased against non-complementary bases at specific positions (e.g. −2 Guanine is not complementary to the crRNA repeat, but is selected against), and prefers non-complementary bases at others (e.g. −5 Guanine is preferred). These preferences support a protein-mediated type IV-A PAM recognition mechanism that does not rely on base pairing with the crRNA repeat, similar to the dsDNA targeting type I CRISPR system (47).

DNA binding by a crRNA-guided Csf-complex will displace the non-target strand forming an R-loop (Figure 7A). In type I CRISPR systems, the Cas3 helicase-nuclease is recruited to the R-loop formed by the Cascade complex. Once loaded onto the non-target DNA strand Cas3 uses metal-dependent ATP binding and hydrolysis to unwind and translocate in a 3′-5′ direction from the target site, while the nuclease domain degrades displaced ssDNA (58–60). The data presented here suggest that CasDinG may play a similar role in type IV-A systems. Like Cas3, CasDinG is an ATP and metal-dependent DNA helicase. Although CasDinG unwinds with the opposite polarity as Cas3 (5′-3′ instead of 3′-5′), the helicase activity of CasDinG would still allow CasDinG to travel from the site of Csf complex targeting along the dsDNA (Figure 5B). All mutations that impaired *in vitro* CasDinG helicase activity also impaired *in vivo* type IV-A immunity, suggesting the immune system relies on helicase activity for proper function, and consistent with recent work by Guo *et al.* demonstrating that gene silencing by the *Pseudomonas oleovorans* type IV-A system appears to deplete RNA transcripts away from the site of Csf complex targeting (8). While it is possible that ATP binding and hydrolysis could regulate a yet-to-be-discovered CasDinG function, these lines of evidence strongly suggest that CasDinG ATP-dependent DNA translocase activity coupled to dsDNA unwinding and/or displacement of RNA primers is an essential activity to type IV-A immunity (Figure 7).

We demonstrated that the accessory N-terminal, vFeS, and arch domains are essential to type IV-A immunity (Figure 4E). Recombinant protein expression and *in vitro* assays indicated that the removal of the vFeS cluster domain and mutation of the vFeS loop decreased protein expression and stability, while the removal of the arch impaired ssDNA binding and helicase activity. Consistent with these observations, biochemical work with XPD and Rad3 helicases from the same family as CasDinG demonstrated that mutation of the FeS cluster and arch domains disrupts helicase activity (43,61,62). Notably, these mutations did not disrupt ssDNA-dependent ATPase activities, indicating both the FeS and arch domains in XPD play important roles in unwinding but are not required for ATP binding or hydrolysis. Our results are consistent with a mechanism where the arch is important for unwinding and the vFeS is important in protein stability, reinforcing the conclusion that dsDNA helicase activity is essential to type IV-A immunity.

Removal of the N-terminal domain did not impair *in vitro* helicase or ATPase activity but strongly disrupted type IV-A immunity. An essential N-terminal domain fused to a helicase motor is reminiscent of the type I CRISPR system. However, unlike the HD domain of the Cas3 helicase, the N-terminal domain of CasDinG does not appear to house a nuclease activity. Rather, we predict the domain adopts a compact fold observed in transcriptional regulators, with a positive surface charge that could bind dsDNA.

In some helicases, accessory domains that interact with nucleic acid have been observed to regulate helicase function. For example, the N-terminus of the Spliceosomal Ski2-like helicase Brr2 impairs ATPase activity unless bound to the U4/U6 snRNA (63). Similarly, our mutagenic studies suggest the N-terminal and arch domain may regulate ATPase activity, as the removal of these domains increased the ATPase activity of CasDinG (Figure 5B). Considering the N-terminal domain is predicted to bind nucleic acid, it is possible that interactions between the N-terminal domain and nucleic acid substrates could modulate any putative regulatory functions.

The predicted N-terminal domain structure also aligns with the DndE protein involved in the phosphorothioation of bacterial DNA in a newly discovered bacterial defense system (53,54,64,65). Putative nickase activity of supercoiled plasmid was observed with recombinant DndE protein (53), suggesting the tantalizing possibility of a similar nickase activity harbored in the N-terminal domain of CasDinG. However, the amino acid sequences of DndE and CasDinG N-terminal domain share little conservation (Figure 6.), and the putative DndE nickase active site has yet to be identified. Thus, these structural insights suggest that the N-terminal domain interacts with dsDNA, but the essential role of the domain in type IV-A immunity remains unknown. We expect that a more clear understanding of the role of the CasDinG N-terminal domain will be revealed when structures and biochemistry of CasDinG in association with the Csf complex and dsDNA targets become available.

## Supplementary Material

gkad546_Supplemental_File

## Data Availability

The NGS data from the PAM depletion assay were deposited to NCBI GEO under the accession number GSE211446. The model coordinates and structure factors for the CasDinG structure have been deposited in the Protein Data Bank under PDB code 8E2W.
