# Supplementary material for: CasDinG is a 5′-3′ dsDNA and RNA/DNA helicase with three accessory domains essential for type IV CRISPR immunity
Source: Nucleic Acids Res. 2023 Jul 3;51(15):8115–32. doi: 10.1093/nar/gkad546 (PMC10450177; doi:10.1093/nar/gkad546)
Supplement: gkad546_Supplemental_File [file gkad546_supplemental_file.pdf]

Supplementary Data

CasDinG is a 5'-3' dsDNA and RNA/DNA helicase with three accessory domains essential for type IV CRISPR immunity

Hannah Domgaard[1], Christian Cahoon[1†], Matthew J. Armbrust[1†], Olive Redman[1], Alivia Jolley[1], Aaron Thomas[2], Ryan Jackson[1,*]

[1]Department of Chemistry and Biochemistry, Utah State University, Logan, Utah, United States
[2]Center for Integrated Biosystems, Utah State University, Logan, Utah, United States

[†]These authors contributed equally to this work

* To whom correspondence should be addressed. Tel.: +1-435-797-1635; Fax: +1-435-797-3390; Email: ryan.jackson@usu.edu

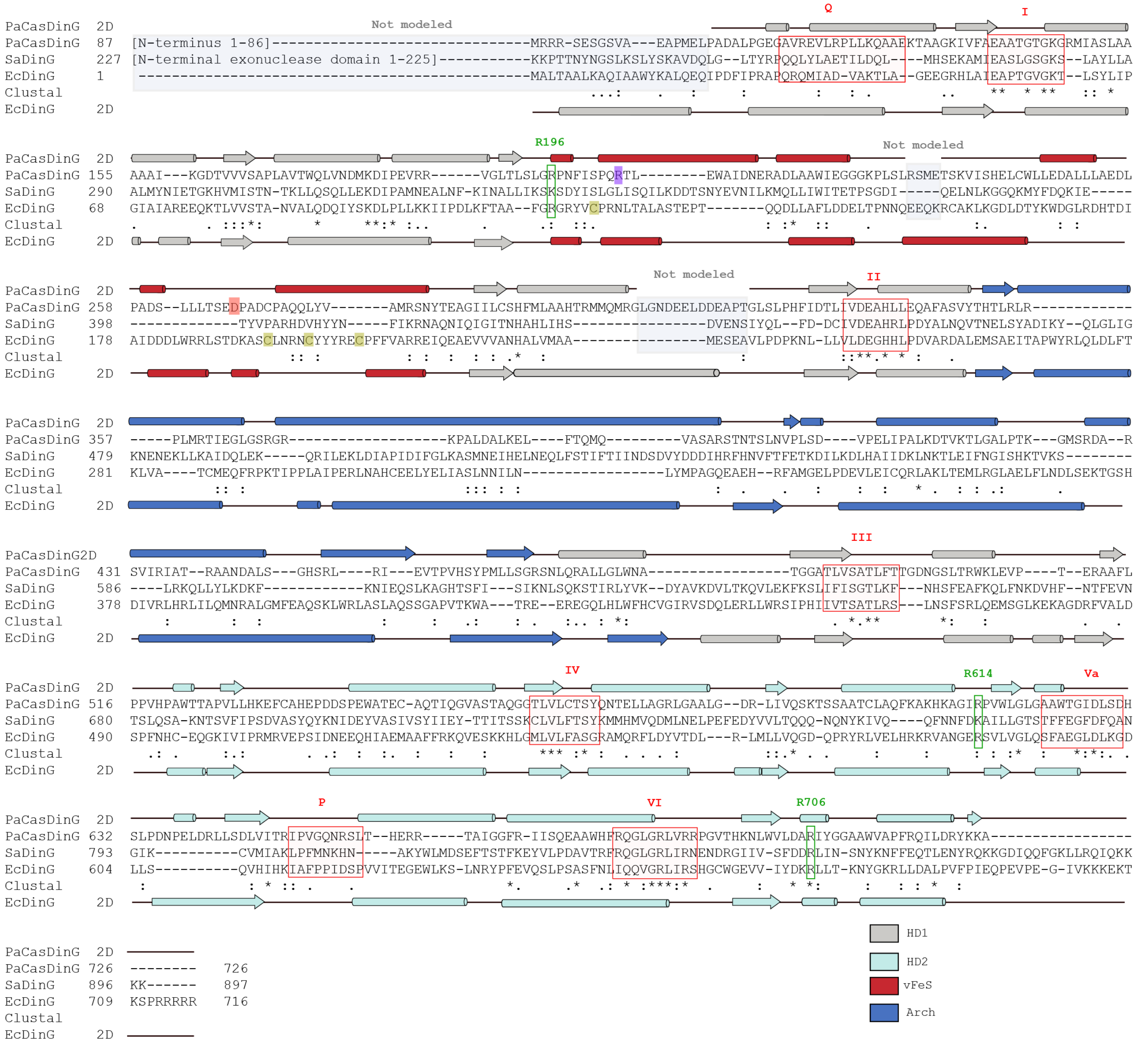

Supplementary Figure S1. Multiple sequence alignment of *Pseudomonas aeruginosa* CasDinG, *Staphylococcus aureus* DinG, and *Escherichia coli* DinG. Secondary structures of both *Pseudomonas aeruginosa* 83 (Pa83) PaCasDinG and *Escherichia coli* (Ec) EcDinG are plotted above and below the sequence alignments, respectively. Conserved helicase motifs are highlighted in red. Cysteines involved in coordinating the FeS cluster in EcDinG are highlighted in yellow. PaCasDinG residues, R204 and D269, are highlighted in purple and orange respectively. These residues form a salt bridge in the vFeS cluster that may compensate for the lack of FeS coordination. Conserved residues linked to XPD mutant disease states in humans (R196, R614, and R706) are indicated in green. The sequence not modeled in the CasDinG crystal structure is highlighted in gray.

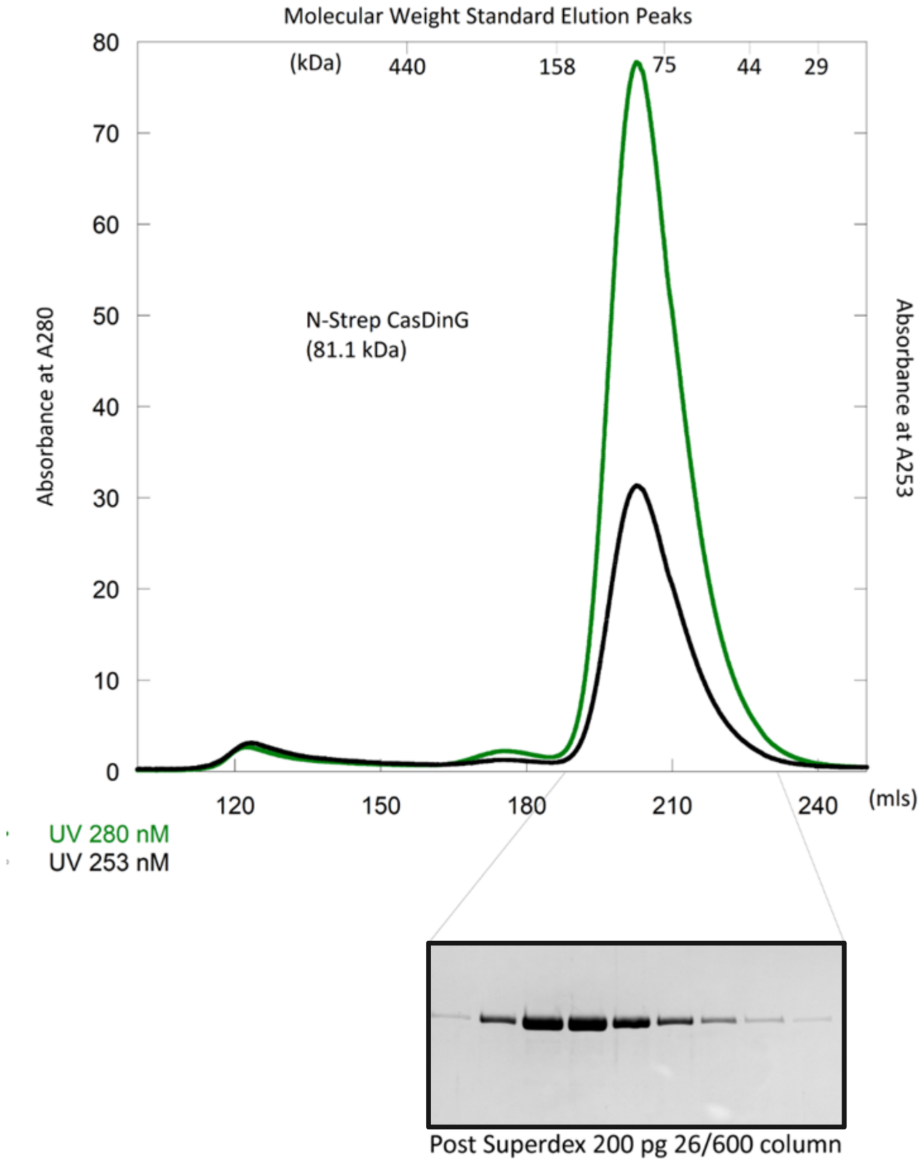

Supplementary Figure S2. Purification of CasDinG. Size exclusion chromatogram of N-terminally Strep tagged CasDinG over a Superdex 200 pg 26/600 column with the associated SDS-PAGE gel depicting protein purity.

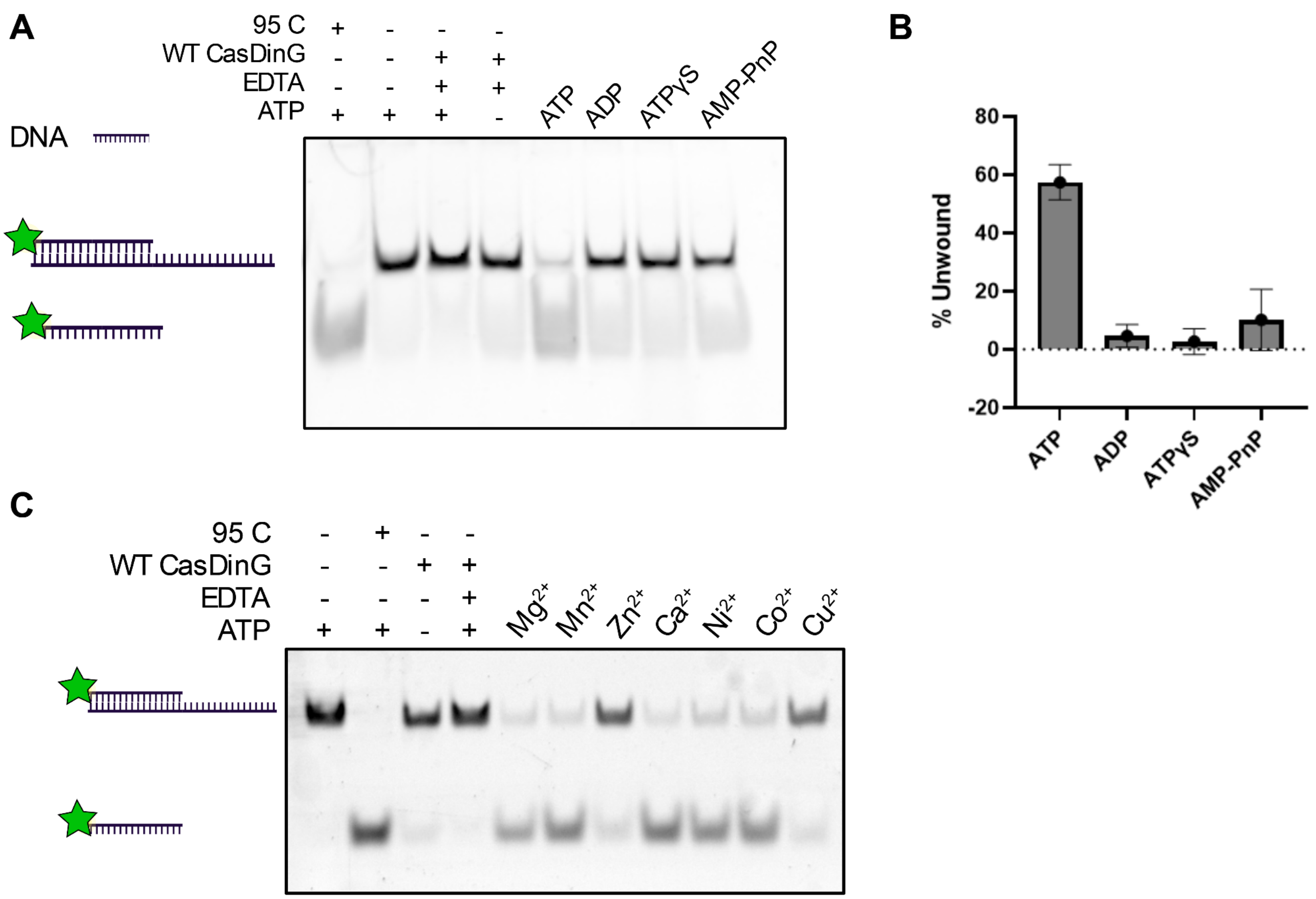

Supplementary Figure S3. Helicase assays using ATP analogs and divalent metals. (**A**) The unwinding of 5' FAM labeled DNA overhang duplex and ATP analogs. Native PAGE shows substantial unwinding only with ATP. (**B**) % Unwound graph showing in-gel densitometry analysis of helicase assays using ATP and analogs with a 5' FAM labeled DNA overhang duplex substrate. Only helicase reactions using ATP had higher than 20% duplex unwound. (**C**) Helicase assay using 5' FAM labeled DNA overhang duplex and various divalent metals. Native PAGE shows unwinding with Mg^{2+}, Mn^{2+}, Ca^{2+}, Ni^{2+}, and Co^{2+}.

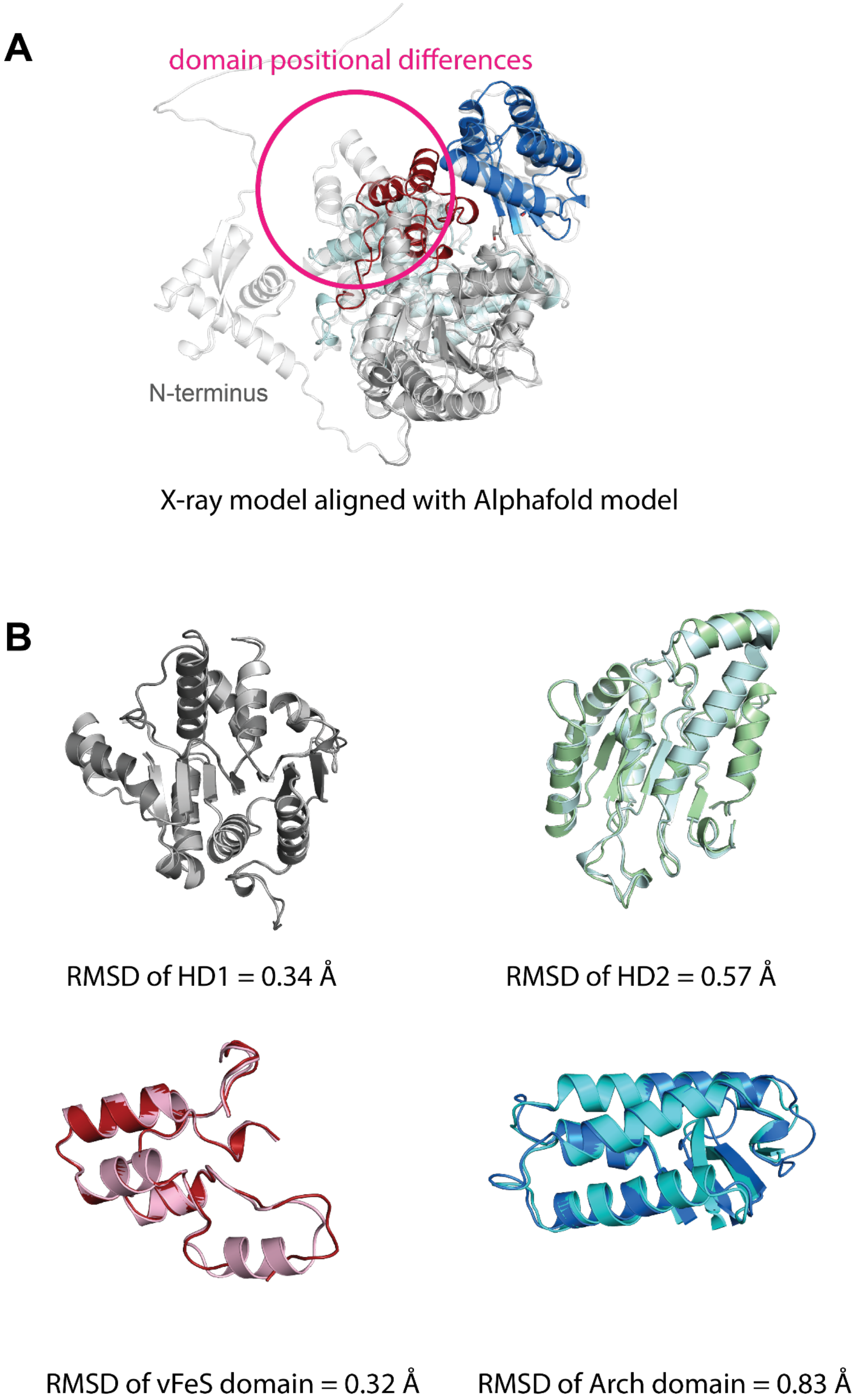

Supplementary Figure S4. Structural alignment of X-ray model and Alphafold structure prediction model. (A) Alignment of the x-ray crystal model and the Alphafold2 model reveals differences in vFeS domain position. (**B**) Alignments of the HD1, HD2, vFeS, and Arch domains of the x-ray model and Alphafold2 prediction model, all alignments show RMSD of less than 1 Å.

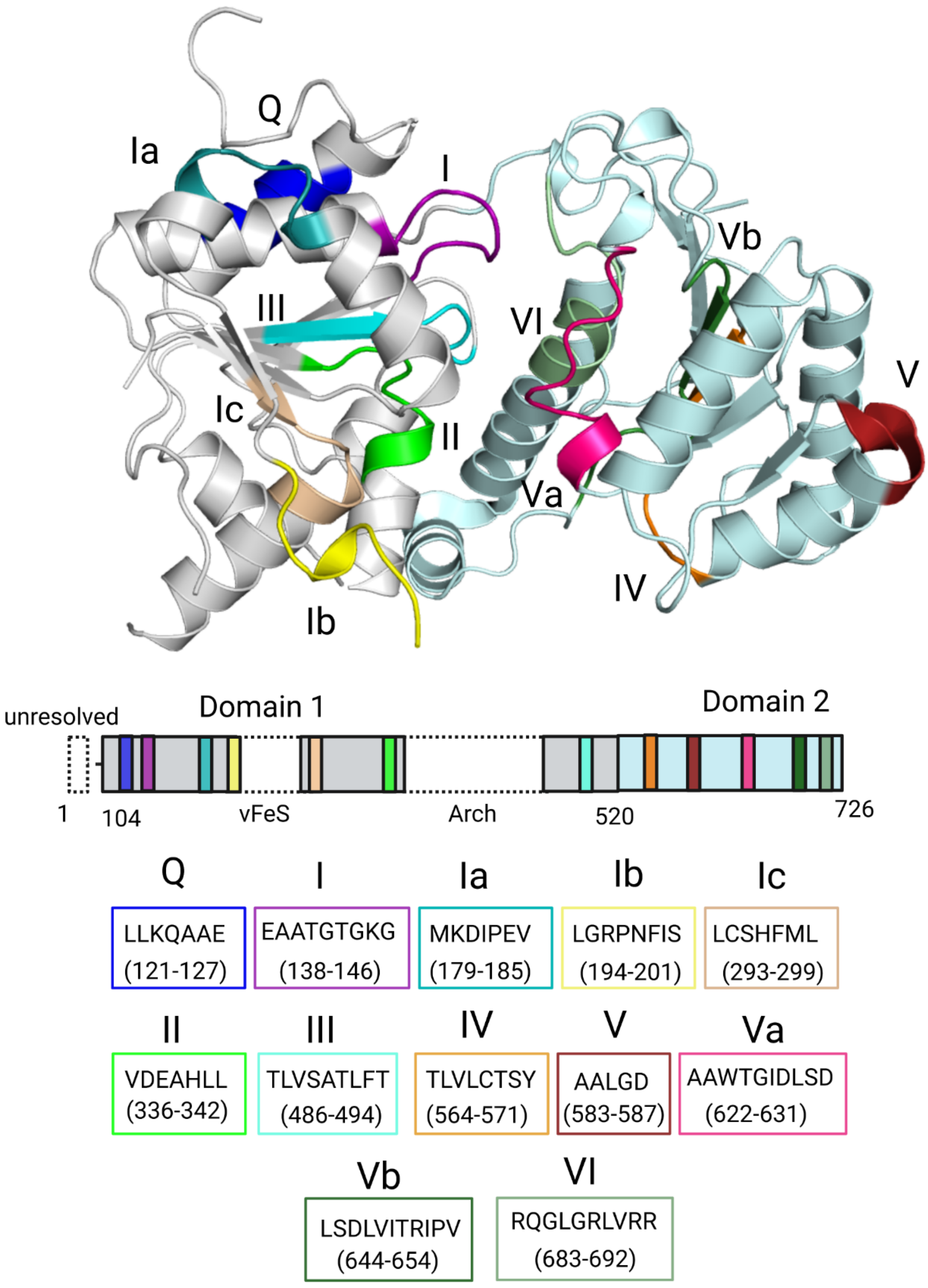

Supplementary Figure S5. Helicase motifs of CasDinG. Motifs Q, I, Ia, Ib, Ic, II & III are located within the helicase domain 1 whereas motifs IV, V, Va, Vb & VI are located in helicase domain 2.

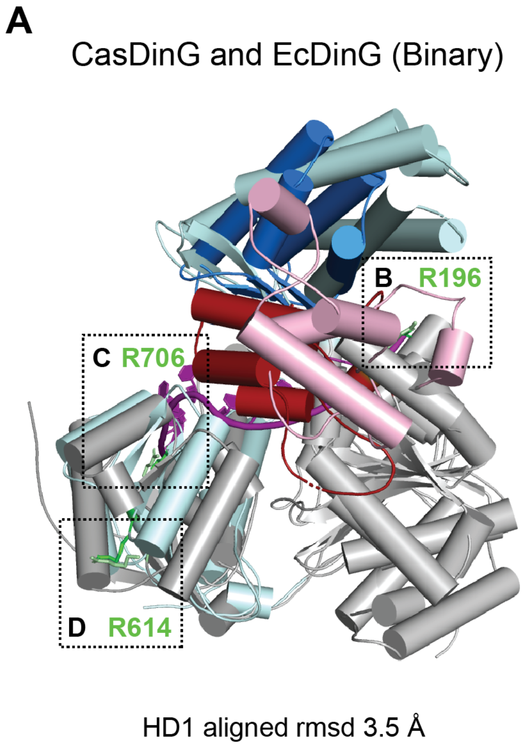

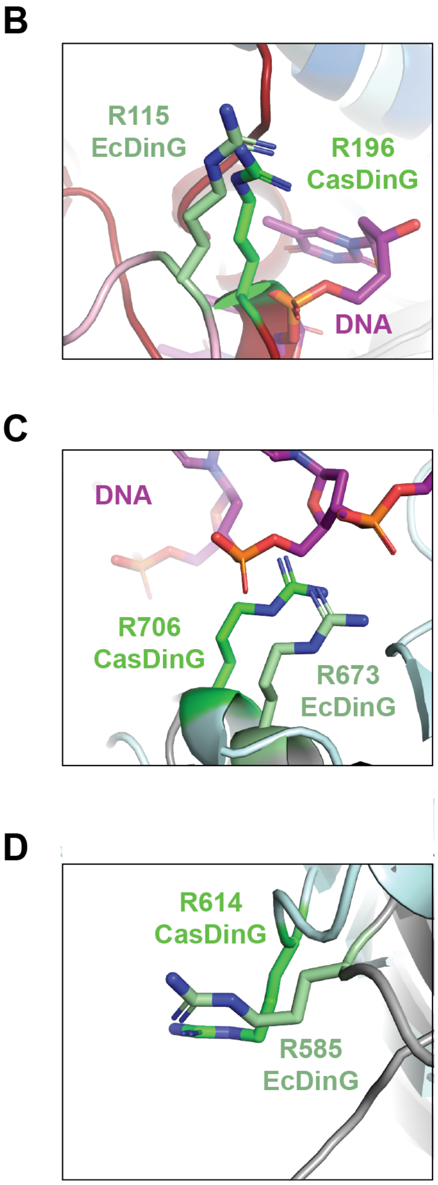

E

Human XPD mutation	Disease	EcDinG residue	CasDinG residue
R112H	TTD	R115	R196
R683W/Q	XP	R673	R706
R592P	TTD	R585	R614

Supplementary Figure S6. Structural Conservation of Residues Linked to Human Disease. (A) Structural alignment CasDinG with the binary structure of EcDinG (PDB 6FWR). Conserved residues linked to disease that are located outside of the conserved helicase motifs are colored green. **(B-D)** Zoomed-in views of the indicated residues. CasDinG is colored in bright green, EcDinG is colored pale green. **(E)** Table indicating human XPD mutations associated with trichothiodystrophy (TTD) and xeroderma pigmentosum (XP) and conservation with EcDinG and CasDinG.

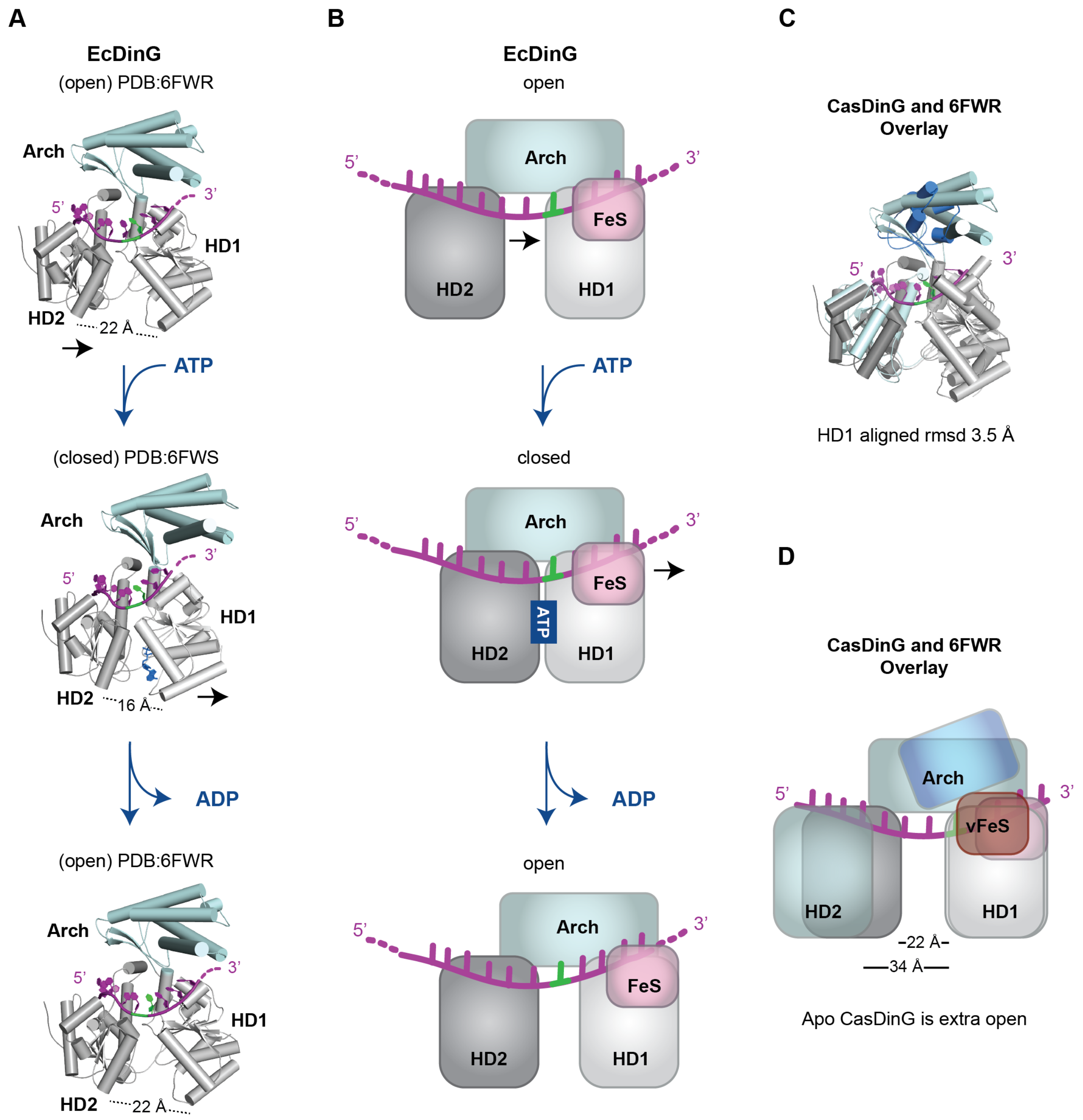

Supplementary Figure S7. Proposed DinG unwinding mechanism. (A) Recent structures of binary (EcDinG and ssDNA PDB:6FWR) and ternary EcDinG (EcDinG, ssDNA, and ADP-BeF PDB:6FWS) display the proposed two-step translocation mechanism where HD1 and HD2 affinities for DNA change during cycles of ATP binding and hydrolysis. The base highlighted green moves past HD1 and HD2 after 1 cycle. FeS domain is removed from structures to visualize HD motions. **(B)** Displayed is a cartoon schematic of the mechanism proposed in A. **(C)** HD1 domains of EcDinG and CasDinG were aligned to create an overlay of binary EcDinG with CasDinG **(D)** A cartoon schematic of the EcDinG overlay indicating how the HD1 and HD2 domains of CasDinG are in an “extra open” conformation compared to EcDinG.

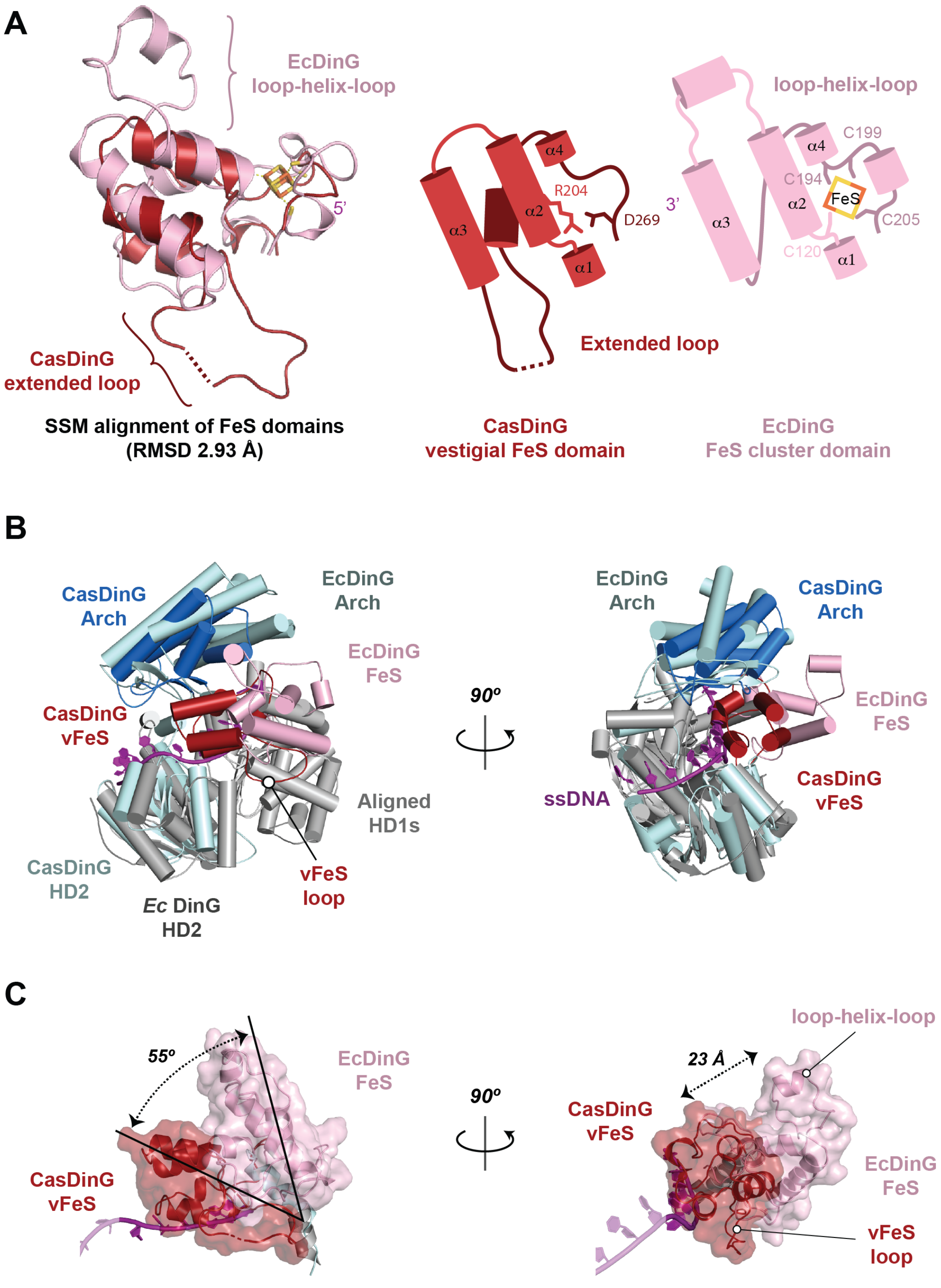

Supplementary Figure S8. FeS domain alignments between CasDinG and *E. coli* (Ec)DinG. (**A**) SSM alignment of the FeS domains highlight the lack of Fe atoms in the CasDinG structure as well as the presence of an extended loop between α3 and α4. (**B**) Global arrangement of domains of CasDinG and the binary EcDinG structure (PDB:6FWR) after an alignment with only the HD1 domains. (**C**) Views of the relative positioning of the vFeS domain and FeS domain when CasDinG is aligned with EcDinG along the HD1 domains. The CasDinG vFeS domain is rotated about 55° and 23 Å away from the position of the EcDinG FeS domain in the binary structure, indicating DNA binding will cause a conformational domain in the vFeS domain.

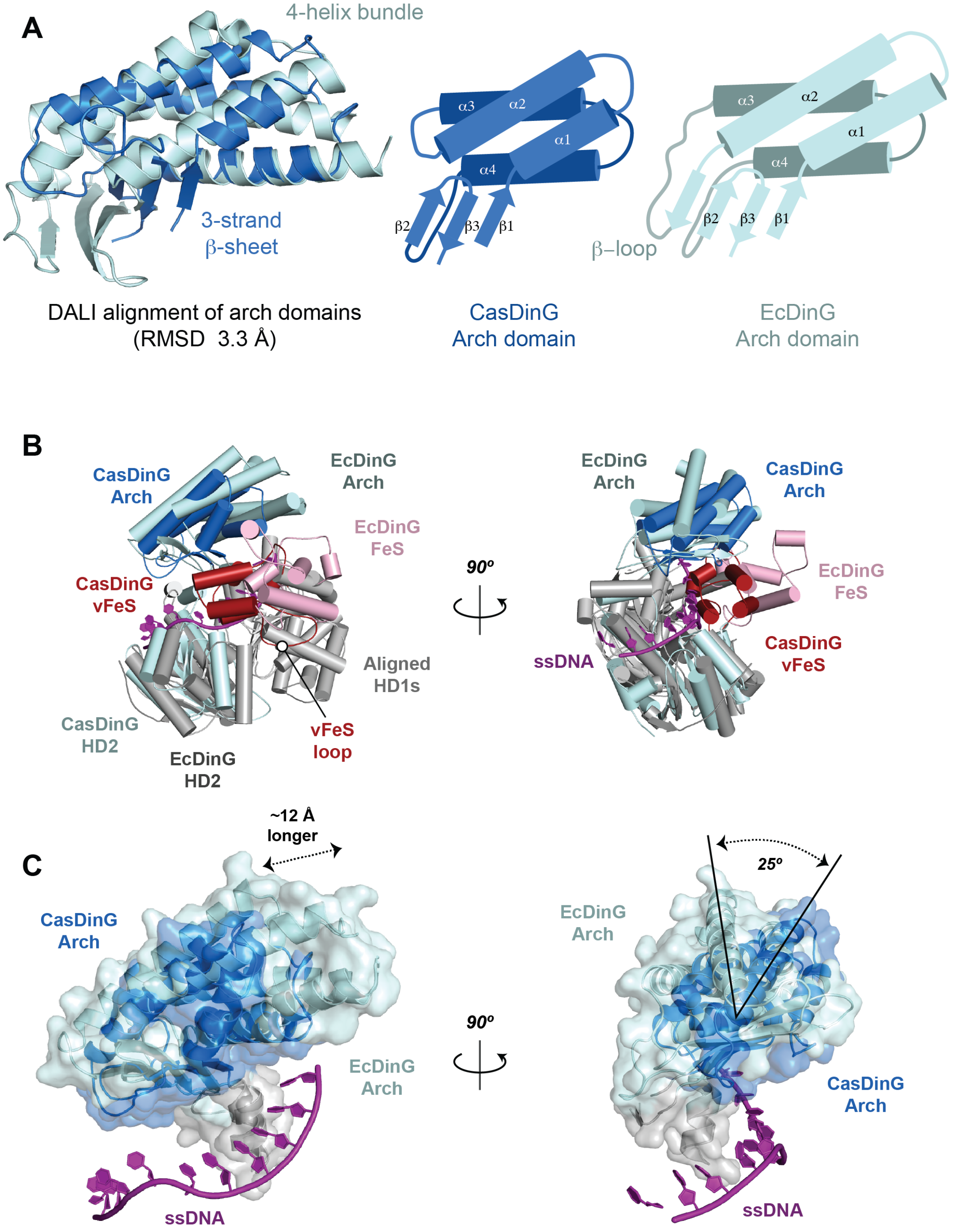

Supplementary Figure S9. Arch domain alignments between CasDinG and *E. coli* (Ec)DinG. (**A**) DALI pairwise alignment of the Arch domains highlights the lack of a β-loop connecting α2 and α3 in the CasDinG structure. (**B**) Global arrangement of domains of CasDinG and the binary EcDinG structure (PDB:6FWR) after alignment of the HD1 domains. (**C**) Zoomed in views of the aligned CasDinG and EcDinG Arch domains. Measurements indicating the larger size and rotated position of the EcDinG compared to CasDinG Arch are indicated.

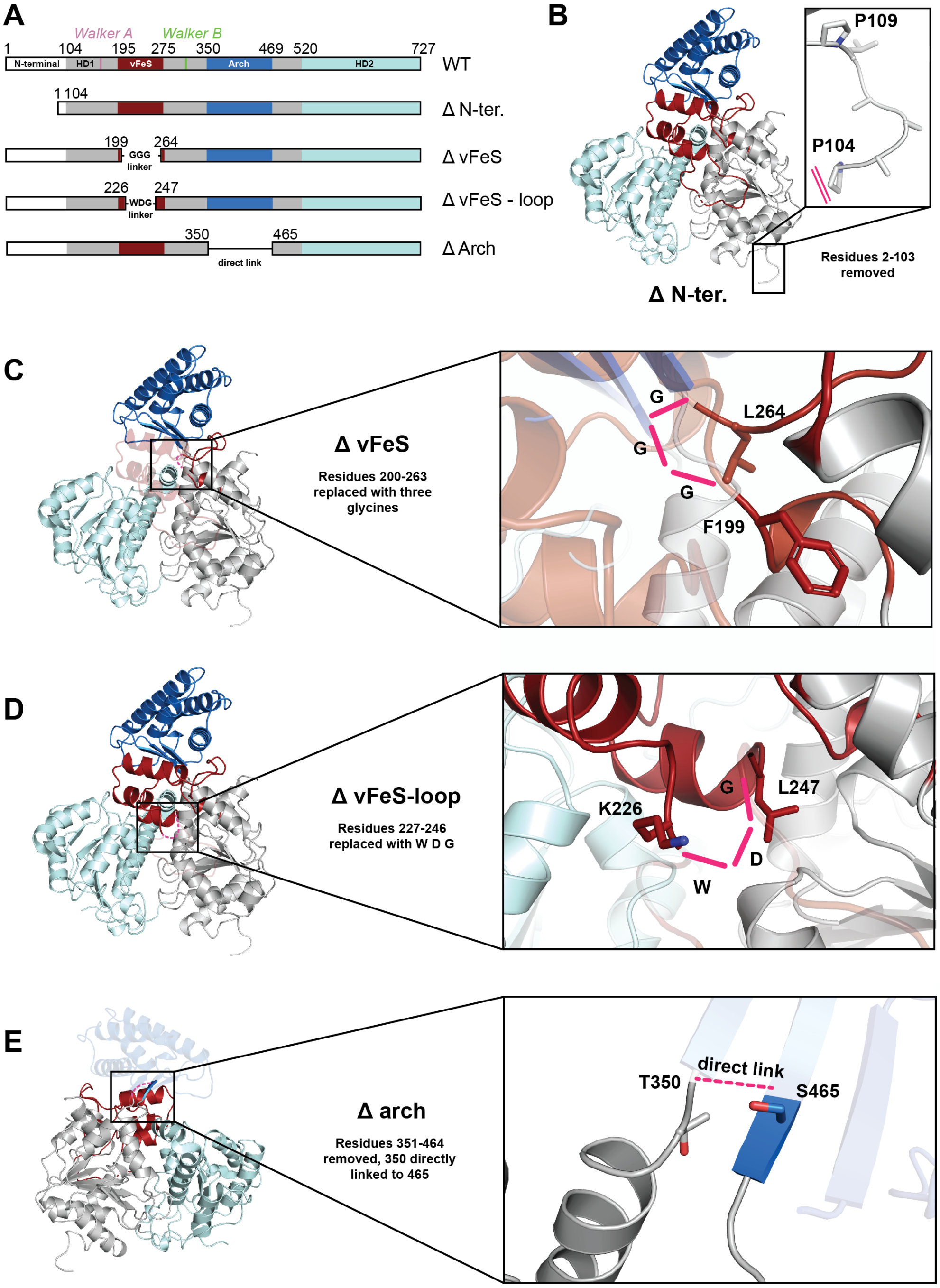

Supplementary Figure S10. Domain mutant design. (**A**) Schematics of the protein sequences for WT, domain deletion mutants, and the vFeS-loop mutant. (**B**) Structure-guided design of the N-terminal deletion. (**C**) Structure-guided design of the vFeS domain deletion mutant. The pink dotted line indicates how F199 and L263 are connected by three glycines in the mutant. (**D**) Design of the vFeS-loop mutant. Residues 227 – 246 were replaced with the amino acids WDG found in EcDinG at the same position in the FeS domain (**E**) Design of the Arch deletion mutant where T350 and N465 are directly linked.

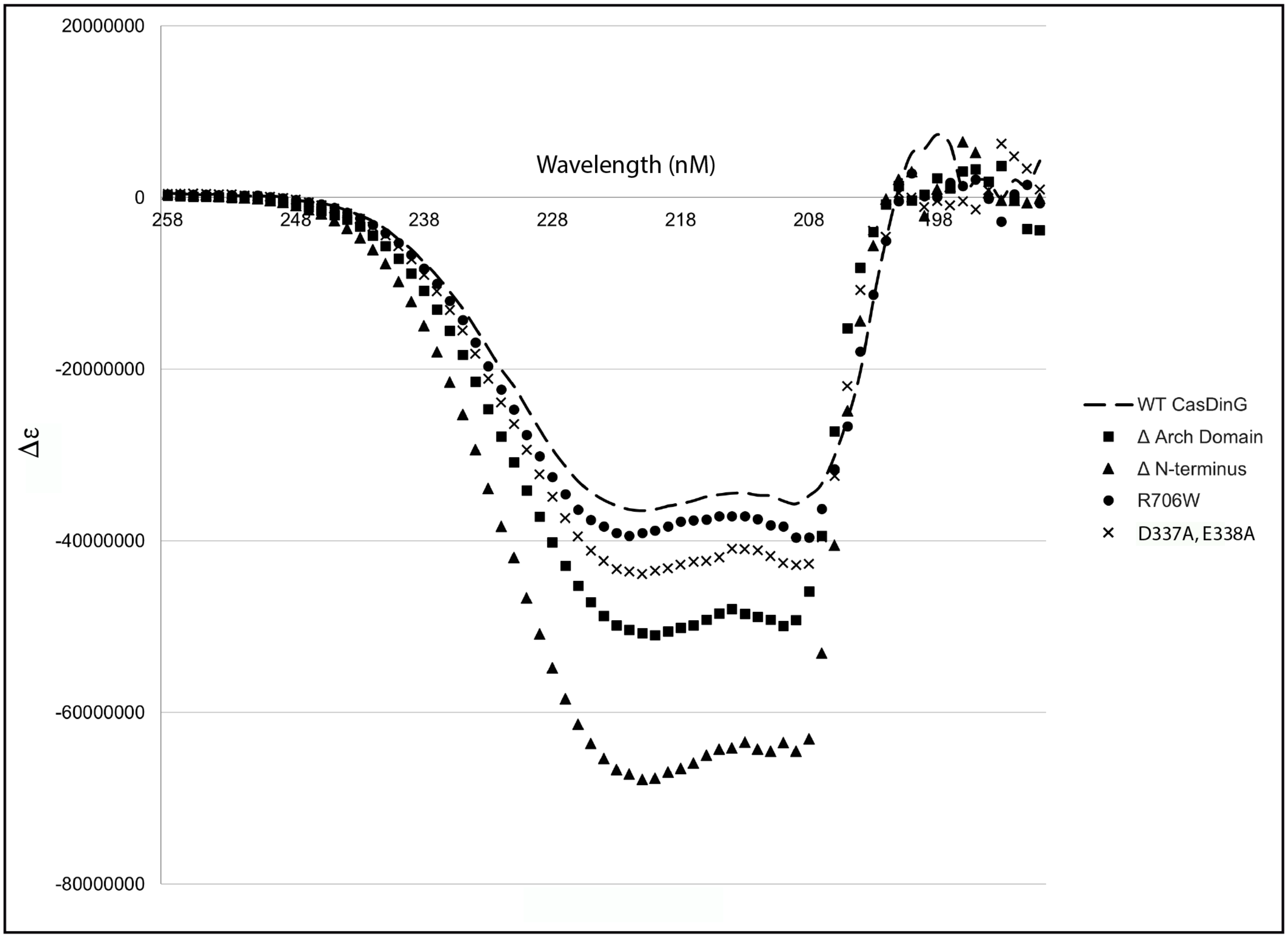

Supplementary Figure S11. Circular Dichroism of CasDinG Mutants

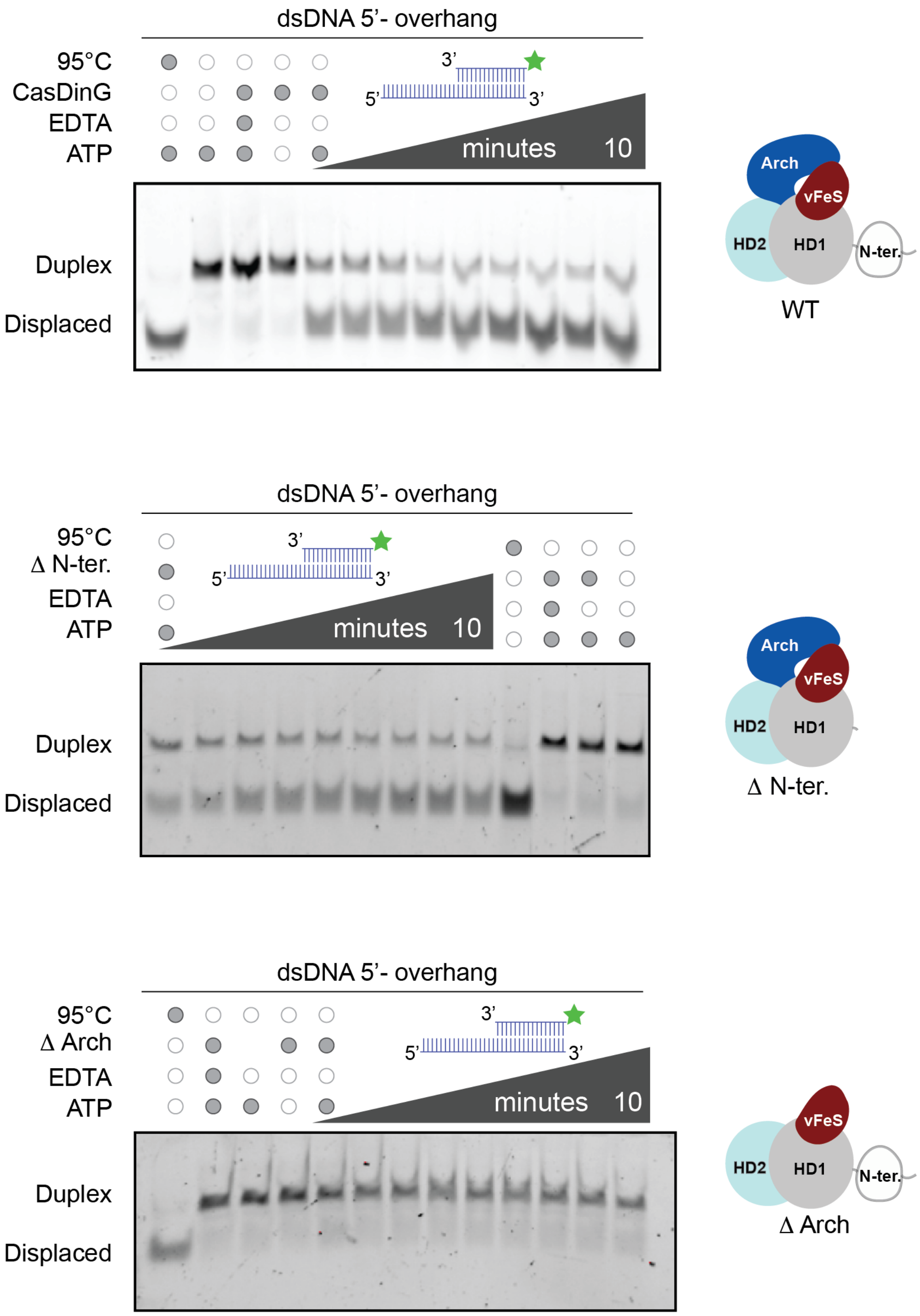

Supplementary Figure S12. Helicase assays with CasDinG domain deletion mutants. Representative unwinding time courses with a 5' overhang duplex substrate added to wild-type (top), N-terminal domain deletion (middle), or Arch domain deletion CasDinG proteins. To the left of the native gel are cartoon diagrams of each construct. Four controls were run with each time course that include a sample heated to 95°C, a sample containing EDTA to chelate activating Mg^{2+} cation, a sample lacking CasDinG, and a sample lacking ATP.

#	DNA RNA	Sequence	Length (nt)	Comp lement	Purpose
1	DNA	5'-TCGTCACCAGTACAAAC-3'	17 nt	2, 3, 4	ssDNA (17 nt) dsDNA 5'- and 3'- overhang (short)
2	DNA	5'-GTTTGTACTGGTGACGA-3'	17 nt	1	dsDNA blunt
3	DNA	5'-TTTTTTTTTTTTTTTTGTTTGTACTGGTGACGA-3'	33 nt	1	dsDNA 5'-overhang (long)
4	DNA	5'-GTTTGTACTGGTGACGATTTTTTTTTTTTTTTT-3'	33 nt	1	dsDNA 3'- overhang (long)
5	RNA	5'-UCGUCACCAGUACAAAC-3'	17 nt	3, 6	ssRNA (17 nt) dsRNA/hybrid 5'- overhang (short)
6	RNA	5'-UUUUUUUUUUUUUUUUGUUUGUACUGGUGACGA-3'	33 nt	1, 5	dsRNA/hybrid 5'- overhang (long)
7	DNA	5'-TCGTCACCAGTACAAACTACAACGCCTGTAGCATTCCACA-3'	40 nt	---	ssDNA (40 nt)
8	RNA	5'- U*CGUCA*CCAGU*ACAAA*CUACA*ACGCC*UGUAG*CAUUC*CAC*A-3'	40 nt	---	PT-ssRNA (40 nt)

Supplementary Table S1. Oligonucleotides used in helicase, ATPase, and anisotropy assays. The (*) of oligo #8 indicate a phosphorothioate modification on the backbone. When FAM labeled, oligonucleotides were labeled on the 5' end.
